# Anticancer Profile of Rhodanines: Structure–Activity Relationship (SAR) and Molecular Targets—A Review

**DOI:** 10.3390/molecules27123750

**Published:** 2022-06-10

**Authors:** Jacek Szczepański, Helena Tuszewska, Nazar Trotsko

**Affiliations:** Department of Organic Chemistry, Faculty of Pharmacy, Medical University of Lublin, 4a Chodźki Street, 20-093 Lublin, Poland; jaacek.szczepanski.93@gmail.com (J.S.); tuszewska.helena93@gmail.com (H.T.)

**Keywords:** rhodanines, anticancer activity, structure–activity relationship, molecular targets

## Abstract

The rhodanine core is a well-known privileged heterocycle in medicinal chemistry. The rhodanines, as subtypes of thiazolidin-4-ones, show a broad spectrum of biological activity, including anticancer properties. This review aims to analyze the anticancer features of the rhodanines described over the last decade in the scientific literature. The structure–activity relationship of rhodanine derivatives, as well as some of the molecular targets, were discussed. The information contained in this review could be of benefit to the design of new, effective small molecules with anticancer potential among rhodanine derivatives or their related heterocycles.

## 1. Introduction

Malignant tumors are still one of the leading causes of human death worldwide. As reported by the WHO, trachea, bronchus, and lung cancers were the sixth main cause of death globally in 2019 [1]. One of the main tools that is still used to combat this common disease is the small-molecule structure with the highest anticancer activity. Therefore, scientists worldwide are still trying to develop new compounds that could selectively target cancer cells [2,3]. This is confirmed by the fact that in 2021 alone the U.S. Food and Drug Administration approved 17 new drugs to be used as anti-tumor agents, out of a total of 50 that were newly registered. That amounts to 34% of all drugs introduced to medical treatment last year [4].

The rhodanine derivatives are small compounds with a broad spectrum of biological activities; they are used as antimicrobial [5], antiviral [6], antitubercular [7], anti-inflammatory [8], antidiabetic [9], and antitumor agents [10,11,12,13].

In the pharmaceutical market, epalrestat (rhodanine-3-acetic acid) has been marketed in Japan since 1992 for treatment of diabetic complications (peripheral neuropathy). Epalrestat is an inhibitor of aldose reductase, the key enzyme in the polyol pathway of glucose metabolism under hyperglycemic conditions. The good clinical safety profile of epalrestat justified the interest of the researchers in rhodanines as potential drug candidates.

Rhodanines were found to induce apoptosis through the modulation of the Bcl-2 family proteins [14,15] or through the modulation of other key signaling proteins [16,17]. Moreover, rhodanines were also reported to reveal their anticancer activity through the inhibition of the phosphatase of regenerating liver (PRL-3) [18].

Furthermore, 5-benzylidene-3-ethyl-rhodanine, also known as BRT-1, is an active anticancer agent which causes S-phase arrest and affects DNA replication in leukemic cells. BTR-1 activates apoptosis and induces cell death [19]. Some of these molecules could become effective and quite selective anticancer drugs in the future.

Among the reviews that have described the biological activity of rhodanines in the last decade, the anticancer activity was described in subsections of the whole review papers [20,21,22,23]. There is only one review that strictly describes the anticancer activity of rhodanines [24]. The literature for our studies was selected from the period of 2011-January 2022, from the following scientific databases: Scopus (Elsevier), SciFinder (Chemical Abstracts), and PubMed. Research articles, short communications, letters, and reports were considered in our studies. Patents were excluded from this review.

There were certain keywords used for the search: “rhodanine”, “2-thioxothiazolidin-4-one”, and “anticancer activity”. The chemical structures considered in this review were limited only to rhodanine. Other structural analogues or isomers of rhodamine, such as thiazolidine-2,4-dione, 2-iminothiazolidin-4-one, thiorhodanine, isorhodanine, and thiohydantoin, were excluded.

## 2. Rhodanines with Anticancer Properties

Positions 3 and 5 in the rhodanine ring were revealed to be chemically more reactive; this plays a significant role in the design and development of new drug-like molecules [21,22]. To present the information available in the scientific literature about molecules in this group in a more efficient way, we divided them according to the method of substitution into 3-substituted, 5-substituted, and 3,5-disubstituted rhodanine derivatives. The molecules described by scientists so far, depending on the place of substitution in the rhodanine nucleus and the nature of the substituents, tend to show a different degree of antitumor activity. We will try to identify those trends that could help in designing new structures with the highest selectivity and potential anticancer activity in the future.

### 2.1. 3-Substituted Rhodanine Derivatives

**Figure 1:**
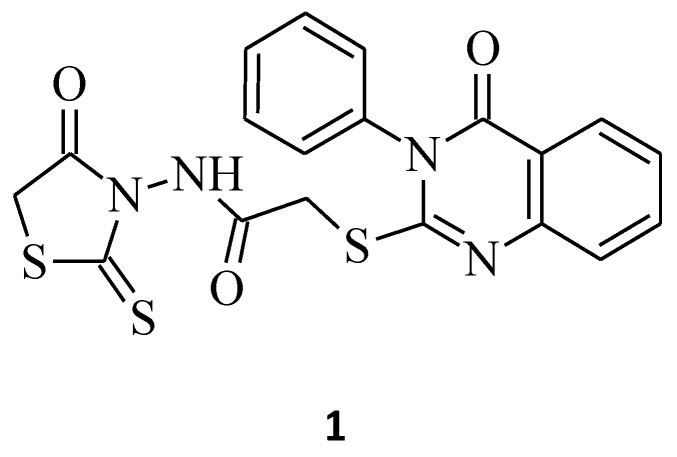
The structure of *N*-(4-oxo-2-thioxothiazolidin-3-yl)-2-[(4-oxo-3-phenyl-3,4-dihydroquinazolin-2-yl)thio]acetamide.

Nguyen et al. synthesized a series of new structures, *N*-(4-oxo-2-thioxothiazolidin-3-yl)-2-[(4-oxo-3-phenyl-3,4-dihydroquinazolin-2-yl)thio]acetamide derivatives, and evaluated them for their cytotoxicity potential against K562 (human chronic myelogenous leukemia) and MCF-7 (human breast adenocarcinoma) tumor cell lines. Compound **1** with the 2-thioxothiazolidin-4-one ring containing the active methylene group (Figure 1), as shown below, exerted moderate cytotoxicity against MCF-7 cells with a % inhibition of cell growth of 64.4% at the concentration of 100 µg/mL [25].

**Figure 2:**
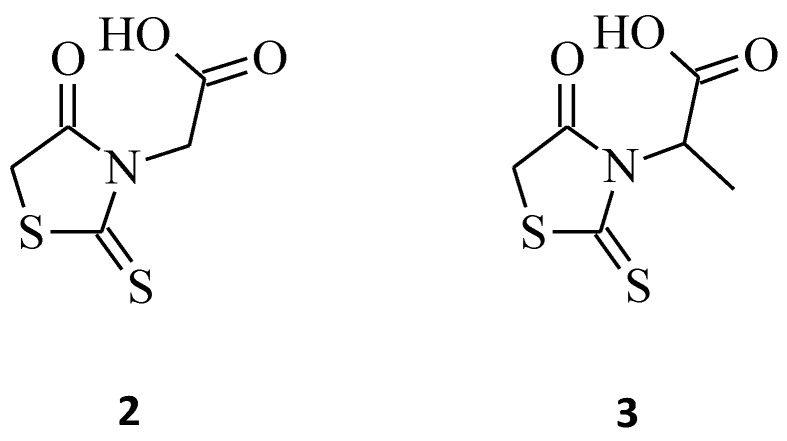
The structures of *N*-substituted rhodanines.

On the other hand, the introduction of small groups such as -CH_2_COOH, -CH(CH_3_)COOH in the *N*-3 position of the rhodanine ring resulted in the formation of the *N*-substituted compounds **2** and **3**, respectively (Figure 2). These molecules showed good antiproliferative activity in the human chronic myelogenous leukemia cell line K562, with an IC_50_ of 14.60, 11.10 µg/mL, respectively, and were twice or three times more potent than the other compounds from the study. Worth noticing is that these compounds were only 3- or 2.3-fold less active in comparison to the reference cisplatin (IC_50_ = 4.78 µg/mL) [26]. The introduction into structure 2 of the methyl group to carboxymethyl moiety only slightly increases the activity. It may have been caused by the similarity of the surface area of the *N*-3 substituent. However, further enlarging the methyl substituent to isopropyl, carboxyethyl, or benzyl substituents into position 3 of the rhodanine ring leads to a 2- or 3-fold decrease in activity.

Furthermore, a structure–activity relationship study was carried out and indicated that, irrespective of the hydrophilic or hydrophobic nature of the groups, the activity decreased with the increase in size [26]. This trend is probably caused by the steric effect that increases with the increasing size of the *N*-substituents.

Moreover, 3-α-carboxyethyl rhodanine **3** was tested for its anticancer activity against the HeLa (human cervical cancer) cell line, and it turned out to be potent with an IC_50_ value of 200 µg/mL (Figure 2) [27].

**Figure 3:**
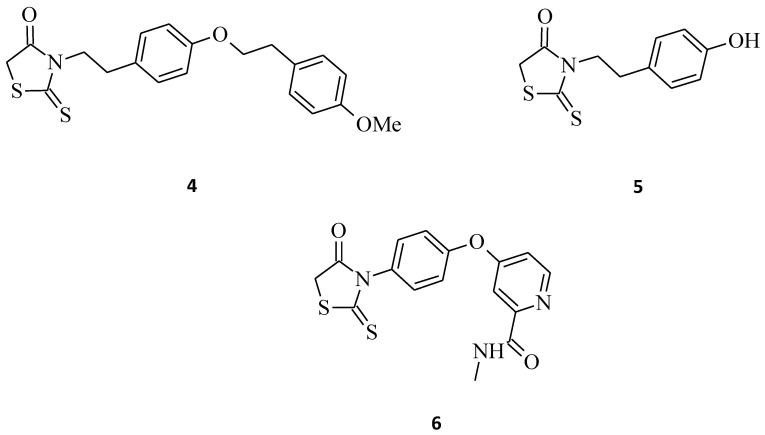
The structures of 3-arylethyl/arylrhodanines.

The antiproliferative activity of the *N*-3-substituted rhodanines was also confirmed by Déliko Dago et al. [28], who evaluated the biological activity of some 3-[4-(arylalkoxy)phenylethyl]-2-thioxo-1,3-thiazolidin-4-one (compound **4**) and 3-[2-(4-hydroxyphenyl)ethyl]-2-thioxo-1,3-thiazolidin-4-one (compound **5**) against representative tumor cell lines (Figure 3). The results of the survival assays showed that 2-thioxo-1,3-thiazolidin-4-one derivative **4** exhibited selective antitumor activity in the colorectal adenocarcinoma HCT 116 cell line, with an IC_50_ value of 10 μM, and did not inhibit the growth of normal fibroblasts (IC_50_ > 25 μM). While compound **5**, interestingly, probably due to the presence of the hydroxyl group and lack of bulky substituents, caused a good increase in the antitumor activities, but without selectivity (MDA-MB231 (breast carcinoma) and HCT 116, IC_50_ 2 μM; Caco 2 (colon adenocarcinoma cells), IC_50_ 3 μM).

**Figure 4:**
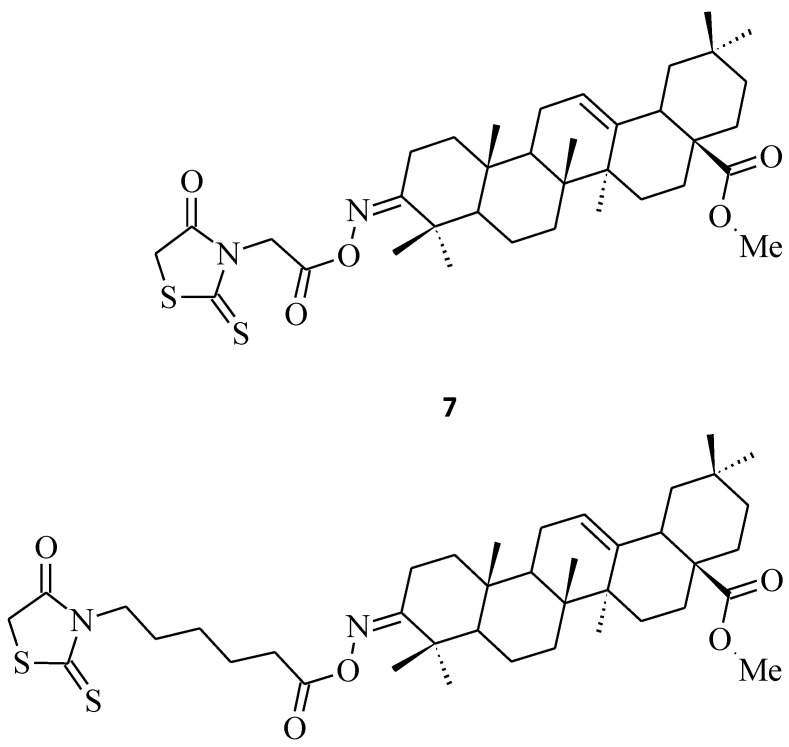
The structures of acylated oximes derivatives of oleanolic acid with 4-thiazolidinone-3-alkylcarboxylic acid moieties.

In the literature, we can also find references to some structures, such as **6** [12], **7**, or **8** [29], that we could classify into this group of *N*-3-substituted rhodanines exhibiting potential antitumor and anticancer activities. Compound **6** (Figure 3) showed some moderate cytotoxicity towards the non-small cell lung cancer line A549, with IC_50_ = 43.6 μM, while compounds **7** and **8** (Figure 4) significantly inhibited the cell growth of certain leukemia and breast cancer cell lines, respectively, with 56.34% and 42.83% line growth at a concentration of 10 µM.

### 2.2. 5-Substituted Rhodanine Derivatives

**Figure 5:**
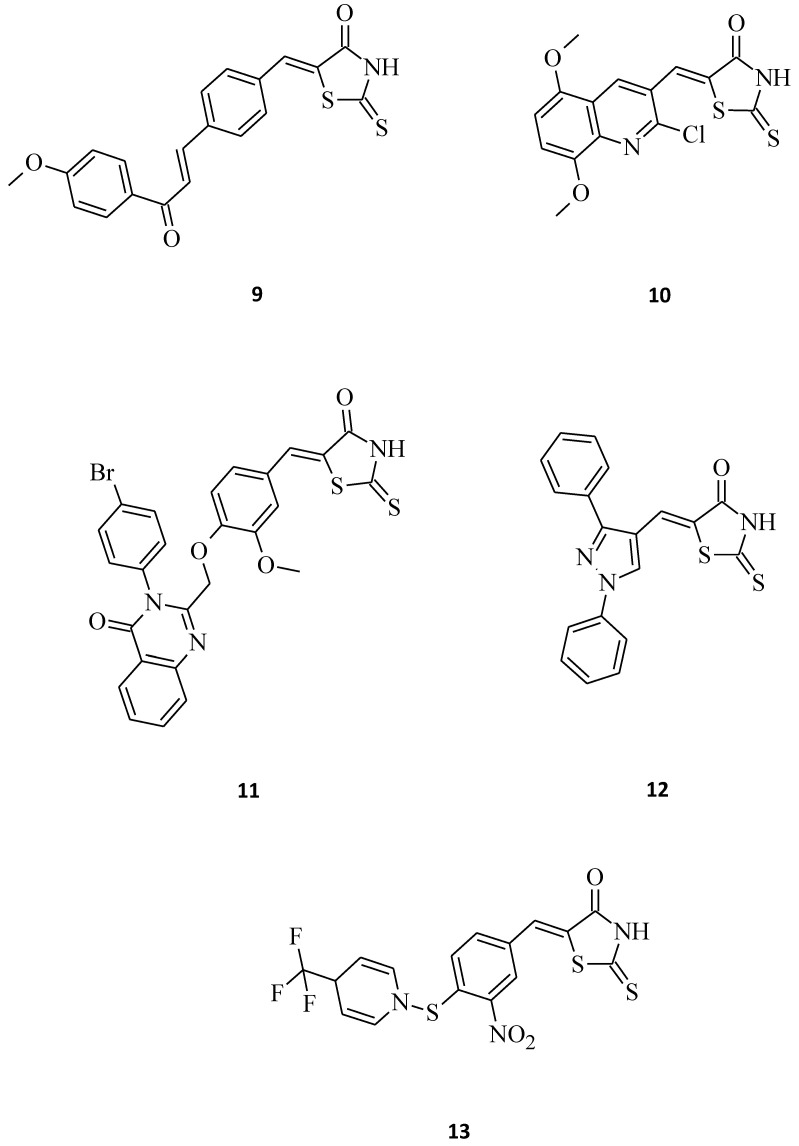
The structures of 5-aryl/heterylmethylidenerhodanines.

Compound **9**, which is 5-{4-[3-(4-methoxy-phenyl)-3-oxo-propenyl]-benzylidene}-2-thioxothiazolidin-4-one (Figure 5), exhibited promising inhibitory activity against the HeLa, HT29 (colorectal adenocarcinomma), A549, and MCF-7 cell lines with the inhibitory concentration (IC_50_) values of 28.3, 24.5, 26.6, and 28.6 μM, respectively [11]. Moreover, 5-((2-chloro-6,7-dimethoxyquinolin-3-yl)methylene) rhodanine derivative **10** (Figure 5) turned out to be potent against the gastric (HGC), prostate (DU-145), and breast cancer (MCF-7) lines [30]. With some further modifications on pharmacophore, compound **10** could serve as a potential anticancer agent, especially towards the DU-145 and HGC cancer cell lines. By comparing the excellent cytotoxic activity of structures **9** and **10** to some previously described 3-substituted derivatives, it can be concluded that the molecules possessing a free -NH-group in the rhodanine moiety seem to be more potent over the N-CH_2_-COOH or *N*-Ph substituted ones. As suggested in the docking studies, this may be connected with the influence of the hydrogen donor group on the active site of the molecular target, such as, for example, the epidermal growth factor receptor (EGFR) [11].

El-Sayed et al. [31] synthesized some novel quinazolinone-based rhodanines that were then biologically evaluated for in vitro cytotoxic activity against the human fibrosarcoma cell line HT-1080 and two human leukemia cell lines, namely HL-60 and K562. Amongst them, structure **11**, bearing a bulky, hydrophobic substituent at the *para* position of the quinazolinone 3-phenyl ring, was the most active, showing cytotoxic activity in the low micromolar range (IC_50_ = 1.2–8.7 µM) towards all the tested cell lines (Figure 5). Its *meta*-substituted counter partners shown in the study were far less active. Interestingly, normal human skin fibroblasts (AG01523) were not affected by this molecule, which indicates that some rhodanines may be selectively toxic against cancer cells. Another great example of a structure that exhibits selective antitumor activity against selected leukemia and non-small cell lung cancer cell lines is **12**. The concentrations of this compound **12** for 50% of the maximal inhibition of the cell proliferation (GI_50_) were tested, and it turned out to be very potent, especially towards the HOP-92 (non-small cell lung cancer), CCRF-CEM (leukemia), and RPMI-8226 (leukemia) cell lines with GI_50_ values of 0.62, 2.50, and 2.52 μM, respectively. The described molecule **12** (Figure 5), as a pyrazole-rhodanine derivative with the LC_50_ > 100 μM indicates the low toxicity of such compounds for normal human cell lines, as required for potential anti-tumor agents [32].

There are also premises in the scientific literature regarding some small molecules that might be fairly useful as a starting point to develop novel anticancer agents. As an example, we can mention structure **13**, which was quite toxic against HeLa and Hep cells, with EC_50_ values of 7.9 and 6.1 μM, respectively (Figure 5) [33].

**Figure 6:**
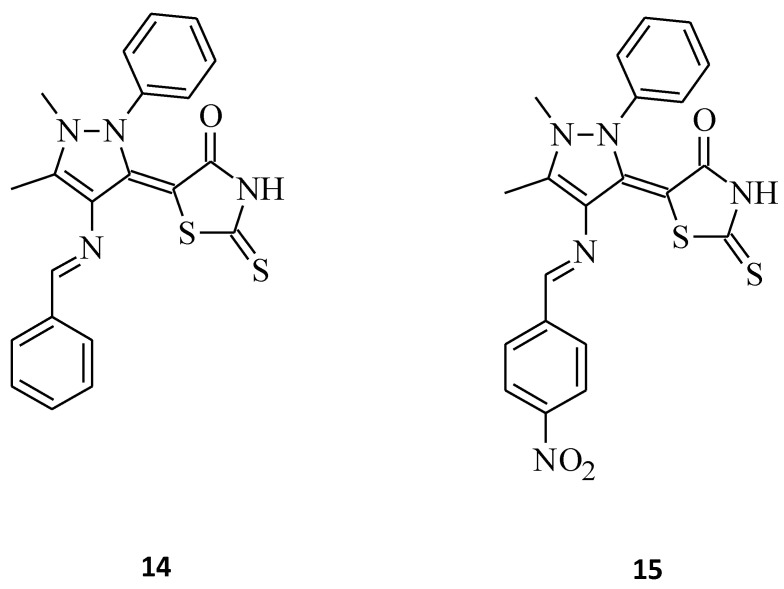
The structures of 5-[4-(phenylmethylideneamino)-1,5-dimethyl-2-phenyl-1*H*-pyrazol-3(2*H*)-ylidene]-2-thioxo-1,3-thiazolidin-4-one (**14**) and 5-[4-({4-nitrophenyl}methylideneamino)-1,5-dimethyl-2-phenyl-1*H*-pyrazol-3(2*H*)-ylidene]-2-thioxo-1,3-thiazolidin-4-one (**15**).

In comparison, El-Mawgoud [34] synthesized some novel 5-[4-(arylmethylideneamino)-1,5-dimethyl-2-phenyl-1*H*-pyrazol-3(2*H*)-ylidene]-2-thioxo-1,3-thiazolidin-4-ones. Compounds **14** and **15** and their cytotoxicity against human breast carcinoma cell line were evaluated (Figure 6). Both of these 5-substituted rhodanines showed high antitumor activity against the cell line MCF-7; however, molecule **14** was more potent than **15** with IC_50_ values of 7.67 μg/mL and 11.7 μg/mL, respectively. This indicates that increasing the mass of the aryl substituent resulted in a decrease in the cytotoxic activity of the tested compound **15**.

**Figure 7:**
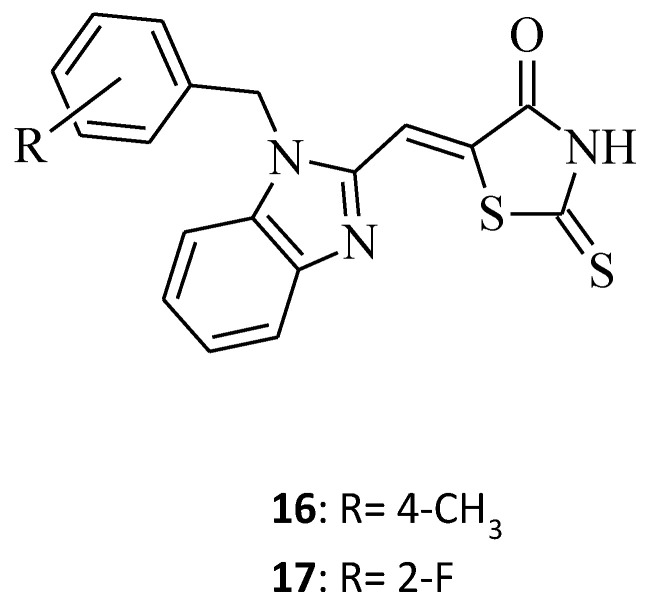
The structure of benzimidazole-rhodanine conjugates.

Some new benzimidazole–rhodanine conjugates, **16** and **17,** were designed, synthesized, and investigated for their cytotoxic activities against human cancer cell lines, including the human acute leukemia cell line (HL-60), the adenocarcinomic human alveolar basal epithelial cancer cell line (A549), the human lymphoma cancer cell line (Raji), and the human breast cancer cell line (MDA-MB-201) [35]. Compound **16**, namely 5-[1-(4-methylbenzyl)-1*H*-benzo[*d*]imidazol-2-yl]methylene-2-thioxothiazolidin-4-one, showed excellent inhibitory activity against tested cell lines, with IC_50_ values of 2.66, 5.31, 4.48, and 6.42 μM, respectively, while the change of the 4-methyl substituent (compound **16**) on the phenyl ring to 2-fluoro for compound **17** resulted in a loss of cytotoxic activity towards all cancer cell lines (Figure 7). This may be related to the fact that compounds with electron donating groups showed better Topo II inhibition than those with electron-withdrawing groups [35].

### 2.3. 3,5-Disubstituted Rhodanine Derivatives

**Figure 8:**
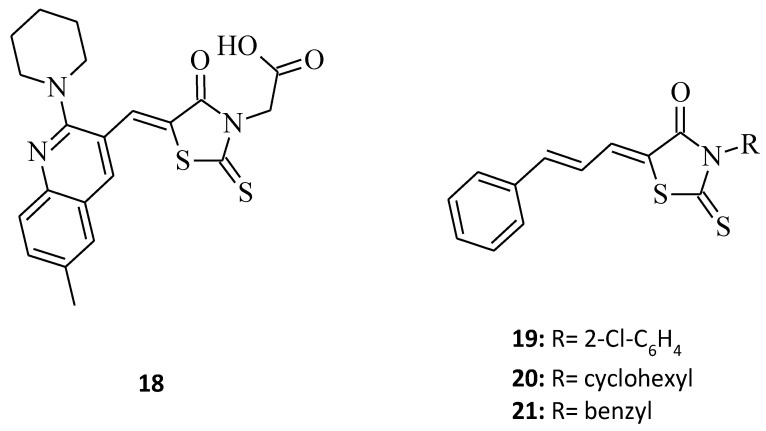
The structures of 3-aryl/alkyl-5-aryl/heterylmethylidenerhodanines.

A new rhodanine analogue bearing 2-piperidine-quinoline scaffold [30], which is compound **18** (Figure 8), was tested on two cancer cell lines, namely the HGC and the MNK 74 (gastric cancer cell line). As with compound **10**, the molecule seems to be effective and hopefully, it will be considered as a potential anticancer agent, especially towards gastric cancer, in the future. In turn, structure **19**, as a 3,5-disubstituted derivative with a cinnamoyl moiety at the fifth position of the rhodanine nucleus, was screened against MCF-7 breast cancer cells [36] and showed some significant anticancer activity, inhibiting the growth of the cancer cell line by 81% at a concentration 10 µg/mL (Figure 8). According to the analogs of the tested compound **19**, shown in the study in [36], the change of the N-3 substitution of the rhodanine ring from 2-chlorophenyl for molecule **19** to 3-cyclohexyl (**20**) and 3-benzyl (**21**) (Figure 8) resulted in the inhibitory decline (inhibitory values of 77% and 71%, respectively). This example indicates a trend, showing that increasing the substituent mass in the third position of the rhodanine moiety improves anticancer activity, as it also does amongst the 3,5-disubstituted rhodanine analogues.

**Figure 9:**
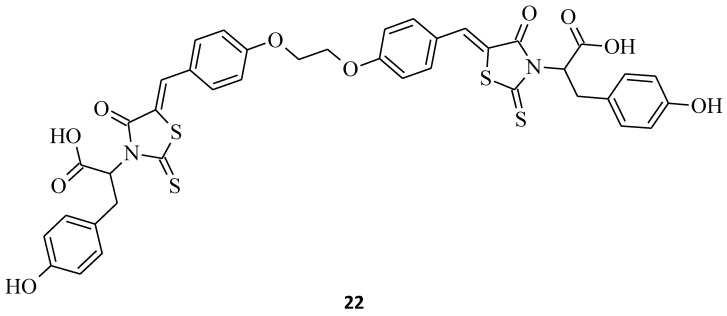
The structure of substituted rhodanine incorporated with tyrosine in the form of a dimer connected via a two-carbon linker.

Prashantha Kumar et al. synthesized a novel rhodanine of biological interest, incorporated with L-tyrosine (compound **22**), and an in vitro cytotoxicity assay against the human lung cancer cell line A549 was carried out [37]. The desired compound **22** turned out to be very effective, with a concentration that inhibited 50% of the growth of A549 cells, with a CTC_50_ (50% of cytotoxicity inhibition) value of 3.6 μg/mL (Figure 9). These results may encourage further investigation of the stereospecific synthesis of other amino acid-incorporated rhodanine derivatives for their anticancer properties.

**Figure 10:**
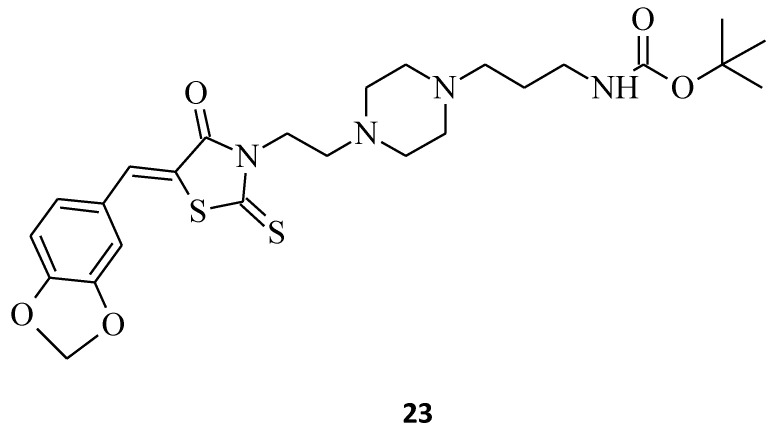
The structure of *tert*-butyl [2-(4-{2-[(2*H*-1,3-benzodioxol-5-yl)methylidene-4-oxo-2-thioxo-1,3-thiazolidin-3-yl]ethyl}piperazin-1-yl)ethyl] carbamate.

New 5-arylidene-2-thioxo-1,3-thiazolidine carbamate, namely compound **23**, was synthesized (Figure 10) [13]. The structure was found to be most active and selective towards the Huh7 D12 (the hepatocellular carcinoma cell line) and Caco2 cancer cell lines, with IC_50_ values of 8 μM, without significant toxicity on normal fibroblasts (IC_50_ > 25 μM).

**Figure 11:**
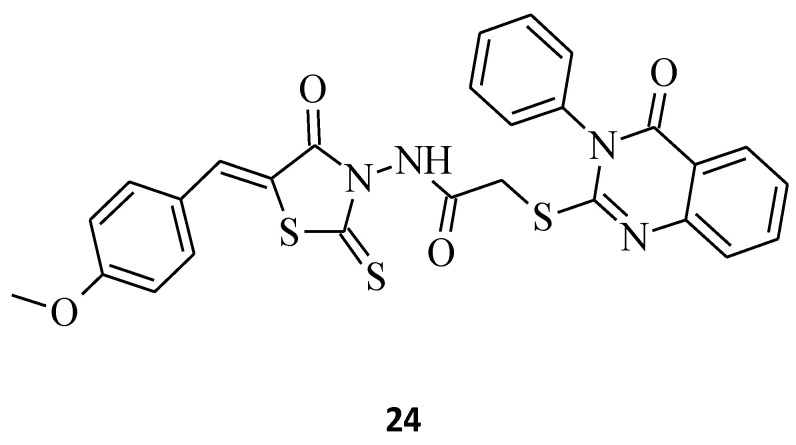
The structure of (*Z*)-*N*-(5-arylidene-4-oxo-2-thioxothiazolidin-3-yl)-2-[(4-oxo-3-phenyl-3,4-dihydroquinazolin-2-yl)thio]acetamide.

*N*-(5-Arylidene-4-oxo-2-thioxothiazolidin-3-yl)-2-((4-oxo-3-phenyl-3,4-dihydroquinazoline-2-yl)thio)acetamide, compound **24** [21], is another good example of the molecule that confirms the relationship trend between structure and its anticancer activity, where 3,5-disubstituted rhodanine derivatives are more suitable for the higher and more selective cytotoxicity against particular cancer cell lines and seem to be more potent towards these cell lines, rather than their *N*-3-substituted counterparts. Compound **24**, with the 4-methoxybenzylidene group introduced at the *C*-5 position of the rhodanine nucleus, inhibited MCF-7 cancer cell line growth by 82.5% at a concentration of 100 µg/mL (Figure 11), whereas its *N*-3-substituted analogue, **1**, only inhibited it by 64.4% (Figure 1). This may suggest that 3,5-disubstituted derivatives represent a better overall profile of a structure with the expected anticancer activity.

**Figure 12:**
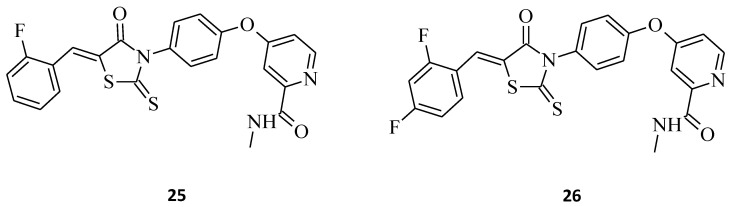
The structures of rhodanine-containing sorafenib analogues.

Novel rhodanine-containing sorafenib analogs were synthesized, namely compounds **25** and **26**, which were then evaluated for their in vitro antiproliferative activity against three cancer cell lines (A549, H460, and HT29) [12]. The results indicate that these structures, especially with **25**, possess antitumor activity superior to the reference drug sorafenib (Figure 12). The most active compound, **25**, with the remarkable IC_50_ values of 0.8, 1.3, and 2.8 μM against A549, H460, and HT29 cell lines, respectively, being *C*-5-(2-fluorobenzylidene) substituted, was much more potent in comparison to the analogue structure **6** (Figure 3). This confirms the conclusion that the level of antitumor activity strongly depends on the substitution pattern of the rhodanine core at the *C*-5 position. While compound **26**, also being much more effective against tested cell lines than **6**, probably due to the more bulky *C*-5 substituent, exhibited lesser antiproliferative activity towards the A549 cancer cell line when compared to **25** (IC_50_ = 3.1 and 0.8 μM, respectively), whereas a similar tendency of the tested compounds **25** and **26** on the H460 and HT29 cancer cell lines is difficult to define. These findings may be a very valuable source of information for designing new rhodanine-based anticancer agents in the future.

**Figure 13:**
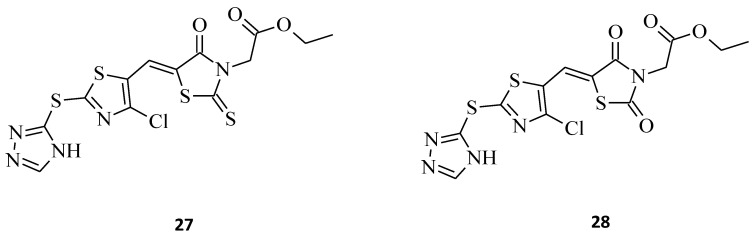
The structures of thiazolyl-rhodanine derivative (**27**) and its thiazolidine-2,4-dione analogue (**28**).

A good example of the superiority of rhodanines over thiazolidinediones is compound **27**, which is a phenyl-substituted triazolothiazolyl-rhodanine derivative [38]. This compound reveals better anticancer properties. This seems to prove that this particular moiety should still be widely researched and used in the development of promising new anticancer agents. The discussed structure showed remarkable cytotoxic activity against two cancer types, namely the hepatocellular carcinoma (HCC) Huh7 and breast cancer MCF-7 cell lines, with IC_50_ values of 4.67 and 2.30 μM, respectively (Figure 13). At the same time, its analogue, **28**, in which the rhodanine moiety was replaced with thiazolidine-2,4-dione, turned out to be non-responsive to the tested cells. It is noteworthy that, according to the results of this study, the lipophilic groups, such as -CH_2_COOC_2_H_5_, introduced on the *N*-3 position of the rhodanine nucleus, may improve the anticancer activity of the compounds and may increase the permeability of the compound to cells. Lipophilic groups may also have a positive impact when implemented into novel rhodanine derivatives as potential antitumor agents, for the same reasons. 

**Figure 14:**
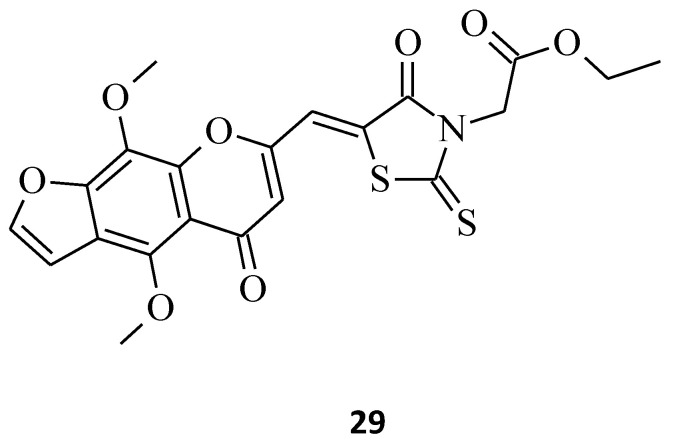
The structure of furochromone derivative.

The next promising rhodanine compound, with a furochromone scaffold in its structure, is structure **29**, which was synthesized and tested for its anticancer properties (Figure 14) [39]. This khellin derivative turned out to be potent on breast cancer cells that originated from different types of tissues, displaying very low EC_50_ values, especially against the MCF-7 and MDA-MB-231 cell lines (EC_50_ = 1.732 and 2.912 μM, respectively). In addition, a superior inhibitory effect of growth on Huh7 cells was observed. Based on this form of furochromone, khellin with a lipophilic rhodanine structure, the discovery of even more active molecules slowing down the progression of the tumor cells could be carried out, mainly for novel anti-breast cancer agents.

**Figure 15:**
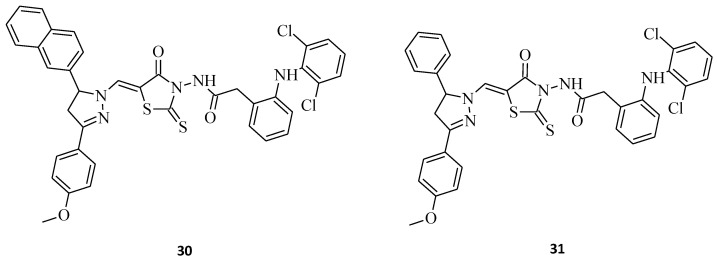
The structures of new rhodanine-pyrazoline hybrid molecules with a diclofenac fragment.

New 5-(3,5-diaryl-4,5-dihydropyrazol-1-ylmethylene)-2-thioxothiazolidin-4-ones with a diclofenac moiety, namely compound **30** and **31** (Figure 15), have been synthesized and evaluated for their antitumor activities [10]. 2-[2-(2,6-Dichlorophenylamino)-phenyl]-*N*-{5-[5-(4-methoxyphenyl)-3-naphthalen-2-yl-4,5-dihydropyrazol-1-ylmethylene]-4-oxo-2-thioxothiazolidin-3-yl}-acetamide, **30**, was found to be the most active structure possessing substantial activity against all tested human tumor cell lines, with average cell growth indices (GPmean) of 22.40%, whereas molecule **31**, being an analogue of **30**, with just a 3-phenyl substitution of the pyrazole moiety instead of 3-naphthalene, was a diametrically weaker agent, with average cell growth indices (GPmean) of 99.30%. These rhodanine-pyrazoline hybrid molecules, with a diclofenac moiety after some further modifications on pharmacophore, could potentially serve as a base for designing novel anticancer drugs.

**Figure 16:**
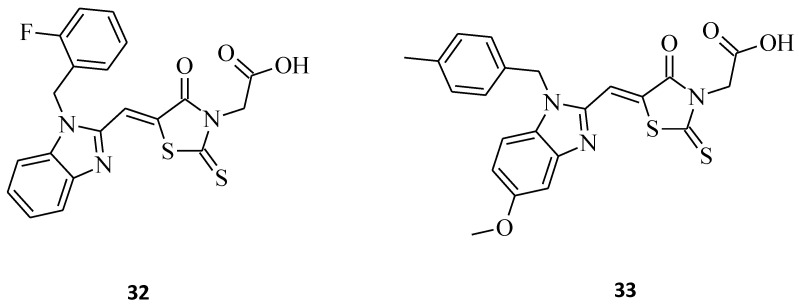
The structures of benzimidazole–rhodanine conjugates.

Benzimidazole–rhodanine conjugates **32** and **33** (Figure 16) were synthesized as analogues to the compounds **16** and **17** (Figure 7), being additionally *N*-3-substituted with acetic moiety [32]. The most potent structure of the discussed compounds was **32**, exhibiting excellent cytotoxic activity against the HL-60, MDA-MB-201, Raji, and A549 cancer cell lines, with IC_50_ values of 0.21, 0.33, 1.23, and 2.67 μM, respectively. The compound was added to the wells at increasing concentrations (0–50 μM). After 48 h, each well was treated with a 20 μL MTT (2.5 mg/mL) solution, and the cells were further incubated at 37 °C for 4 h. In comparison to **17**, it seems that acetic moiety is crucial for the cytotoxic effect, at least for the tested cancer cell lines. It is noteworthy that both of the 3,5-disubstituted rhodanines, **32** and **33**, displayed significantly better activity than their 5-substituted counterparts from the study. The results show that the introducing of acidic moiety, especially acetic one, at the third position of the rhodanine ring may have a significant impact on the potential anticancer activity of the desired compounds.

**Figure 17:**
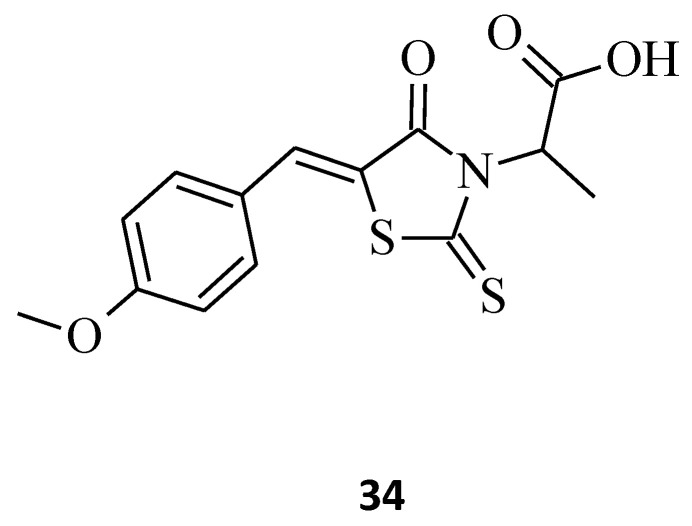
The structure of 5-{[(4-methoxyphenyl)methylidene]-4-oxo-2-thioxo-1,3-thiazolidin-3-yl}propanoic acid.

Another indisputable piece of evidence confirming the superiority of 3,5-disubstituted structures over their 3-substituted rhodanine counterparts, with regard to their anticancer properties, is compound **34**. This 3-α-carboxy ethyl-5-benzylidene rhodanine derivative caused inhibition of HeLa cancer cell growth by 52% (Figure 17), while **3** (Figure 2) was less effective against the tested HeLa cells, with an inhibitory percentage of 14.28% [27]. When comparing these two structures, it is clear that the introduction of 4-methoxy benzylidene moiety for **34** increased its cytotoxicity levels significantly towards the tested HeLa cancer cells.

**Figure 18:**
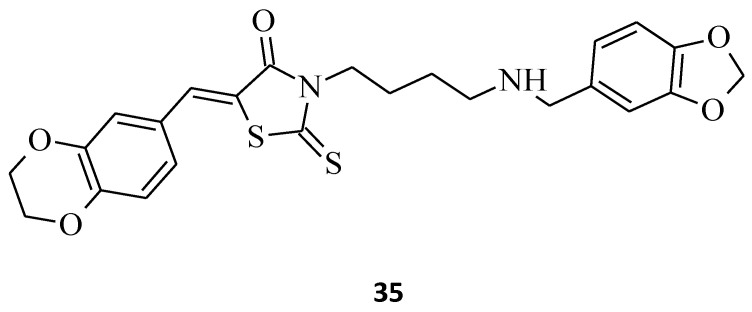
The structure of 3-(4-arylmethylamino)butyl-5-arylidenerhodanine.

Novel 3-(4-Arylmethylamino)butyl-5-arylidene-rhodanine, **35**, was synthesized [40], and its antitumor activity was tested. This structure exhibited promising antitumor effects in the HuH7 D12, HaCat, and MDA-MBD 231 cell lines, with IC_50_ values below 10 μM (Figure 18). It is worth emphasizing that compound **35**, while being potent against cancer cell lines, did not inhibit the growth of normal fibroblasts (IC_50_ > 25 μM).

**Figure 19:**
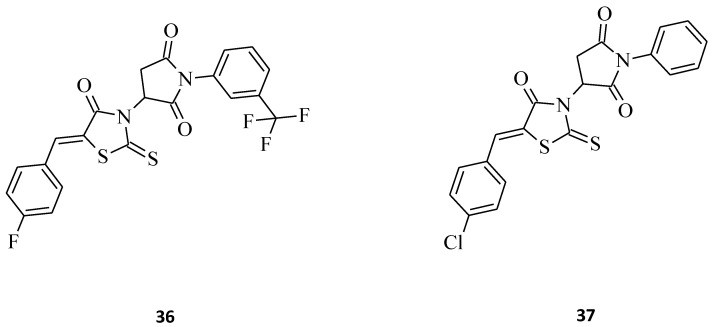
The structures of pyrrolidinedione-thiazolidinone hybrids.

Kryshchyshyn et al. introduced some new pyrrolidinedione-thiazolidinone hybrids, **36** and **37** (Figure 19), and then tested these 5-ylidene-3-(1-aryl-pyrrolidine-2,5-dione)-rhodanines towards selected cell lines for their antileukemic properties [41]. Both compounds inhibited Dami cell line growth by more than 50%, and **36** was the more potent of the two (Dami cell line growth = 35.10%). In turn, structure **37** turned out to be more active against HL-60 cells, with an inhibitory value of almost 60%. Based on the presented data, one could say that compounds **36** and **37** possess satisfactory toxicity levels on leukemia cell lines and might be used for the drug-like molecules.

**Figure 20:**
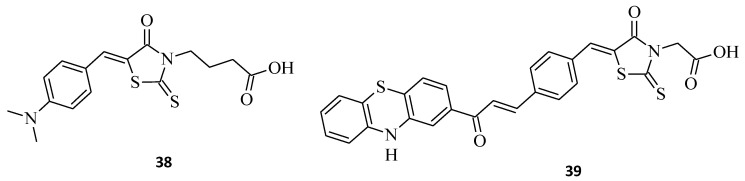
The structures of 5-arylidenerhodanine-3-carboxylic acids.

Selected rhodanine-3-carboxylic acid derivative, **38**, was synthesized and its cytotoxicity against human ovarian carcinoma A2780 and A2780cisR-cells has been determined [42]. Structure **38**, namely 4-[5-(4′-*N*,*N*-dimethylaminobenzylidene)-rhodanine]-butyric acid, displayed excellent anticancer activity, with IC_50_ = 4.4 and 3.3 μM towards both tested cell lines, A2780 and A2780cisR, respectively (Figure 20). Interestingly, the selected compound **38** was much more cytotoxic than cisplatin in both cancer cell lines. Phenothiazine, chalcone, and rhodanine moieties that are pharmacologically active were presented in the hybrid molecule **39** and seem to act synergetically when evaluated for their antiproliferative activity against K562 cancer cell lines (Figure 20) [43].

**Figure 21:**
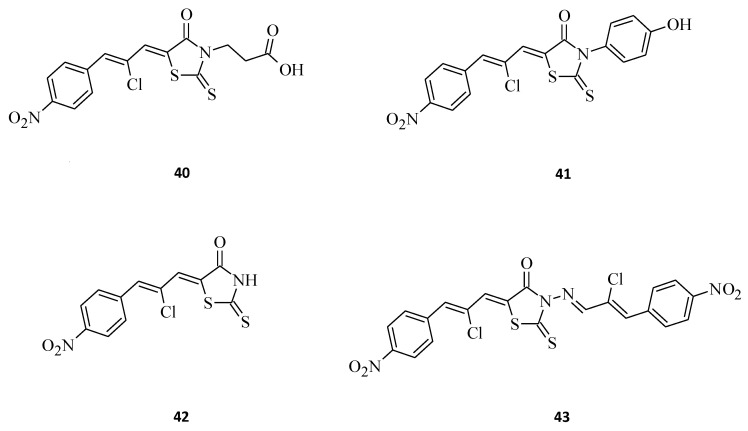
The structures of new ciminalum–thiazolidinone hybrid molecules.

Buzun et al. [44] designed and synthesized a series of new 5-[(*Z*,*2Z*)-2-chloro-3-(4-nitrophenyl)-2-propenylidene]-thiazolidinones, which are a combination of a thiazolidinone core and a structural fragment of the ciminalum, namely(2*Z*)-2-chloro-3-(4-nitrophenyl)prop-2-enal. Ciminalum is an active Gram-positive and Gram-negative antimicrobial factor [45]. Amongst these hybrid compounds, 3-{5-[(*Z*,*2Z*)-2-chloro-3-(4-nitrophenyl)-2-propenylidene]-4-oxo-2-thioxothiazolidin-3-yl}propanoic acid, **40** (Figure 21), displayed the best antimitotic activity, with mean GI_50_ values of 1.57 μM and a certain sensitivity range towards the leukemia (MOLT-4, SR), colon cancer (SW-620), CNS cancer (SF-539), melanoma (SK-MEL-5), gastric cancer (AGS), human colon cancer (DLD-1), and breast cancers (MCF-7, MDA-MB-231) cell lines. Structure **41**, being a p-hydroxyphenyl derivative was also very effective, while the absence of a substituent in the C-3 position of the rhodanine moiety (**42**), or an additional ciminalum fragment (**43**), led to decrease in anticancer cytotoxicity (Figure 21). Both compound **40** and compound **41** had low toxicity levels towards normal human blood lymphocytes and a broad range of therapeutic effects. These data suggest that the presence of a ciminalum moiety in the C-5 position of the 2-thioxo-4-thiazolidinone ring is a very interesting possibility for designing novel and potentially active agents, as high cytotoxicity of the tested 5-[(*Z*,*2Z*)-2-chloro-3-(4-nitrophenyl)-2-propenylidene]-2-thioxo-4-thiazolidinone-3-carboxylic acids against several cancer cell lines have been established.

**Figure 22:**
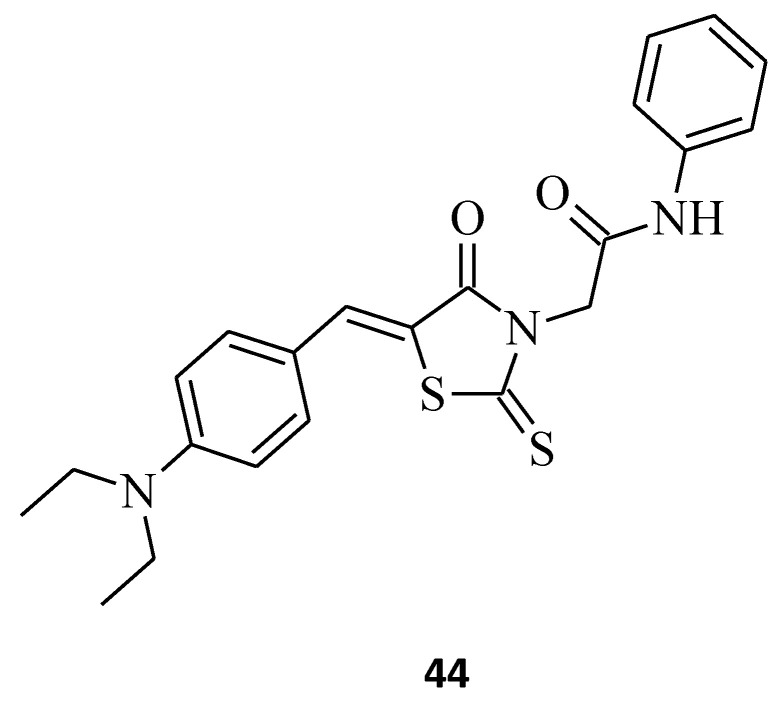
The structure of (*Z*)-2-(5-benzylidene-4-oxo-2-thioxothiazolidin-3-yl)-*N*-phenylacetamide.

Zhou et al. [46] combined the cores of a 2-thioxo-4-thiazolidinone moiety, a, b-unsaturated ketones, and acrylamide derivatives to design new microtubule-interacting agents as potentially active antiproliferative compounds against different cancer cells. (*Z*)-2-(5-(4-(dimethylamino) benzylidene)-4-oxo-2-thioxothiazolidin-3-yl)-*N*-phenylacetamide, **44** (Figure 22), displayed the best antiproliferative activity towards A549 (IC_50_ = 7 μM) cancer cells, comparable to that achieved with gefitinib (IC_50_ = 5.89 μM). Moreover, molecule **44** turned out to be only weakly cytotoxic against NRK-52E cells, with IC_50_ = 14.7 μM, while promoting microtubule protofilament assembly, leading to a reduction in microtubule density and disordered networks. It seems that a bulky steric-hindering moiety at the para position favors the good bioactivity of modified (*Z*)-2–(5-benzylidene-4-oxo-2-thioxothiazolidin-3-yl)-*N*-phenylacetamide derivatives, according to compound **44**. These results might help with developing novel microtubule-stabilizing structures, which are potent in the treatment of cancer.

**Figure 23:**
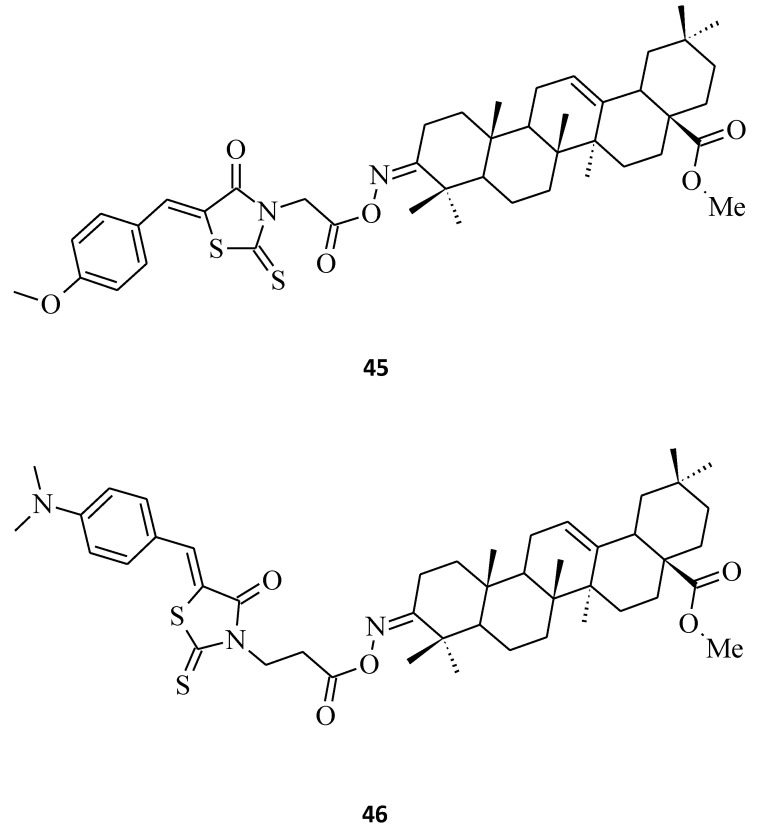
The structures of 3-*O*-acyloleanolic acid derivatives with rhodanine core.

Last, but not least, rhodanine-oleanolic acid derivatives, **45** and **46** [29], had a significant inhibitory effect on some breast cancer (**45**) and ovarian cancer (**46**) cell lines (Figure 23). However, any tendency between the cytotoxic effects for different substituents of these oleanolic derivatives, including **7** and **8** (Figure 4) compounds and cancer cell lines, is difficult to determine.

**Figure 24:**
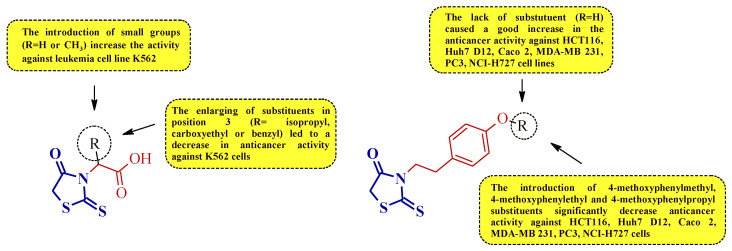
The structure–activity relationship for some 3-substituted rhodanines with anticancer properties against leukemia, colorectal, prostate, breast, hepatocellular, and lung carcinoma cells.

Summarizing the structure–activity relationship analysis, the following trend can be observed. The introduction of small substituents in position 3 of the (2-thioxothiazolidin-3-yl)acetic acid derivatives (compounds **2** and **3**) improves the activity against the leukemia cell line K562. However, the enlarging of the substituents in this position (ex. isopropyl, carboxyethyl, or benzyl) was unfavorable for antiproliferative activity against K562 (Figure 24).

A similar trend was observed for compounds **5** and **4**. Expanding the substituent by the 4-methoxyphenylalkyl groups of compound **5** decreased the anticancer activity against some leukemia, colorectal, prostate, breast, hepatocellular, and lung cancer cell lines (Figure 24).

**Figure 25:**
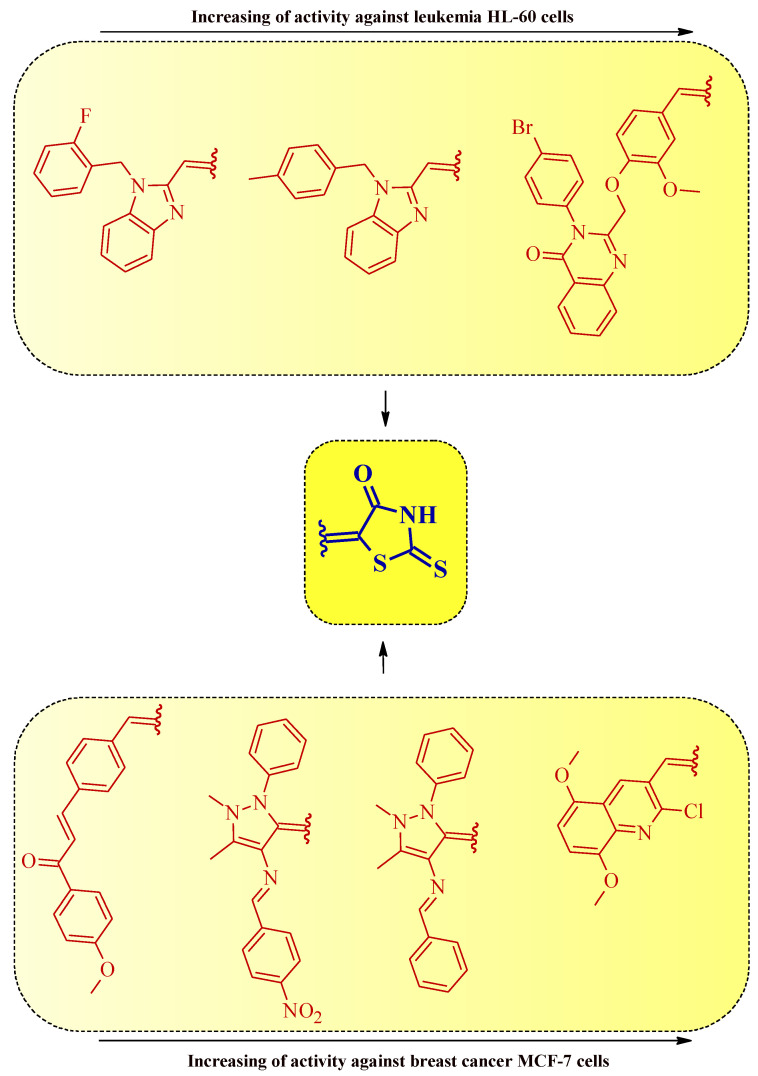
The structure–activity relationship for some 5-substituted rhodanines with anticancer properties against leukemia and breast cancer cells.

It is notable that the presence of heteryl moiety was more preferable for good anticancer activity than aryl substituent in 5-substituted rhodanines (Figure 25).

**Figure 26:**
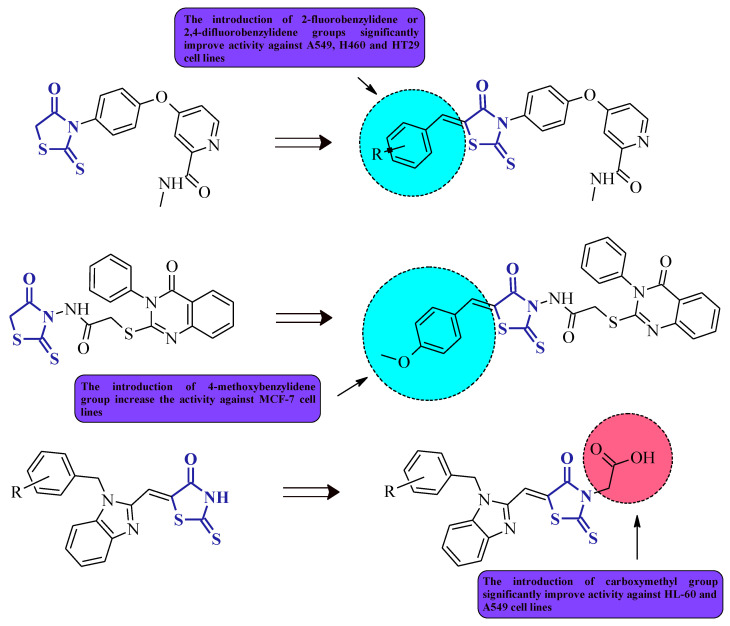
The structure–activity relationship for some 3,5-disubstituted rhodanines with anticancer properties.

It is worth noticing that the introduction of simultaneous substituents at positions 3 and 5 of the rhodanine system generally increases the anticancer activity in comparison with the 3- or 5-monosubstituted rhodanine derivatives (Figure 26).

The information about the activity of the most potent 3-, 5-substituted, and 3,5-disubstituted rhodanine derivatives is summarized and presented in Table S1 (see Supplementary Materials).

## 3. Targets

**Figure 27:**
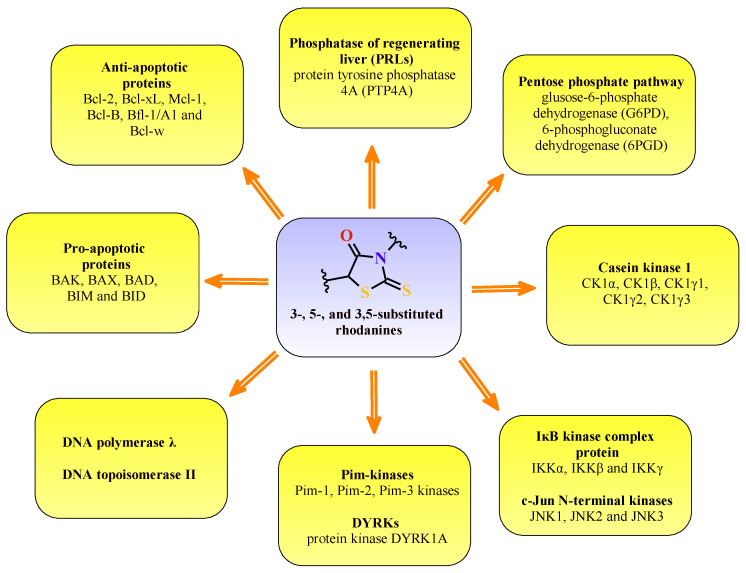
The molecular targets for 3-, 5-, and 3,5-substituted rhodanines.

Protein kinases are an important class of enzymes that regulate various biological processes. These enzymes can catalyze protein-phosphorylation on serine, tyrosine, and threonine residues, which are often deregulated in human diseases. So far, a total of 518 human kinases have been investigated as potential therapeutic targets [47]. That is why the constant search for protein-kinase inhibitors for novel anticancer agents is still a very interesting target, especially in the pharmaceutical industry (Figure 27).

**Figure 28:**
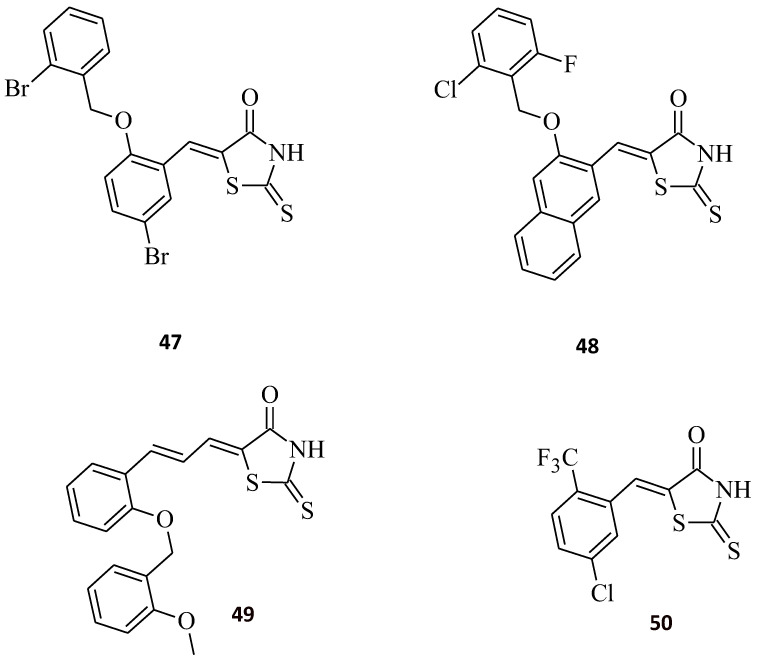
The structures of rhodanine derivatives, potent as PRL-3 inhibitors.

The phosphatases of the regenerating liver (PRLs) family, also described as protein tyrosine phosphatase 4A (PTP4A), are dual-specificity phosphatases possessing multiple cellular functions that are still largely unknown. However, the latest results indicate that PRLs are oncogenic across many different types of human cancers. PRLs are overexpressed in advanced-stage tumors and metastases compared to initial/preliminary stage cancers, and the high expression of the PRLs is usually matched with poor patient prognosis. PRL-3 is the most well-known of the PRLs that have been considered as potential therapeutic targets in cancer [48]. Rhodanine benzylidene derivative **47** and rhodanine naphthylidene derivative **48** were synthesized (Figure 28), and their inhibitory effect against PRL-3 was measured [18]. Compound **47** turned out to be the most active with an IC_50_ value of 0.9 µM as **48** displayed a weaker inhibitory effect towards PRL-3 (IC_50_ = 1.7 µM). The results indicate that the introduction of a benzylidene moiety at C5 of the rhodanine nucleus favors a higher inhibitory potency of PRL-3 over 5-naphthylidene substitution. According to the structural information from the study [49], PRL reveals a strong hydrophobic character, bearing a large entrance; so, it is noteworthy that the introduction of substituents with a hydrophobic nature enhanced the inhibitory effects of rhodanine molecules against PRL-3. The 5-cinnamilidenerhodanine derivative **49** showed a slightly better inhibition activity of PRL-3 than its benzylidene analogue **47** (IC_50_ = 0.8 µM vs. 1.1 µM, respectively) [50]. The 5-[5-chloro-2-(trifluoromethyl)benzylidene]-2-thioxothiazolidin-4-one (**50**) could effectively inhibit PRL-3 with IC_50_ = 15.22 µM. Additionally, compound **50** inhibited expression of PRL-3 and increased the phosphorylation of PRL-3 substrates, as well as decreasing the survival of SW-480 cells (IC_50_ = 6.64 µM), and induced apoptosis. Compound **50** is a promising anticancer PRL-3 targeting drug candidate [51].

**Figure 29:**
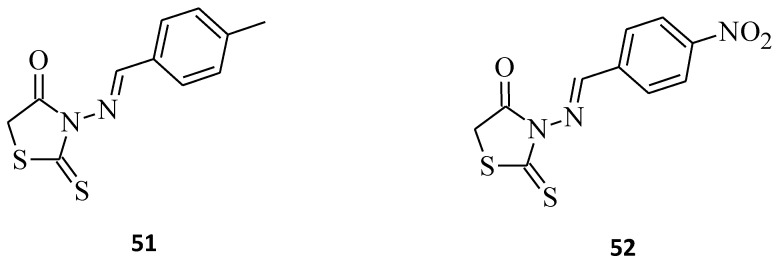
The structures of rhodanines containing benzene moieties as pentose phosphate pathway inhibitors.

The pentose phosphate pathway is a metabolic pathway parallel to glycolysis [52], in which activation of (PPP) enzymes, namely glucose-6-phosphate dehydrogenase (G6PD) and 6-phosphogluconate dehydrogenase (6PGD), significantly affects tumor metabolism by contributing to malignant transformation, enlarging tumor progression, preventing cell apoptosis, and promoting tumor metastasis and angiogenesis [53]. High expression of the PPP, in particular the 6PGD enzyme, has previously been reported mainly in the regulation of multiple human solid cancers, such as leukemia cancer, liver cancer, colon cancer, breast cancer, ovarian cancer, and thyroid cancer. However, the promoting cancer progression mechanism by PPP enzymes is still being studied [54,55]. The inhibitory activities of selected rhodanine derivatives containing different benzene moieties **51** and **52** (Figure 29) on the PPP enzymes G6PD and 6PGD were tested [56]. Structure **51** was found to be most potent against G6PD with IC_50_= 6.54 µM, while **52** displayed a stronger inhibitory effect towards 6PGD, with an IC_50_ value of 10.04 µM. When comparing both structures, it is clear that introducing a nitro group into the *para*-position of a benzene moiety favors the better inhibitory activities of N-3-substituted rhodanines towards the 6PGD (PPP) enzyme, whereas the G6PD enzyme seems to be more prone to the inhibitory effects of N-3-substituted rhodanine derivatives bearing the 4-methylbenzylidene group. The molecular docking studies results showed that the 4-methylbenzylidene moiety (compound **51**) interacted with hydrophobic residues in the catalytic active site of the G6PD enzyme. Compound **51** interacted with Phe171 (key residue of catalytic activity) residues by a closer location into the catalytic active site [57]. Interaction between the compound and Phe171 may lead to inhibition of the enzyme by interfering with the interaction between the residue and the substrate. Several G6PDs have demonstrated similar modes of interaction [58,59]. On the other hand, the binding modes showed that compound **52** with the 4-nitrobenzylidene group may inhibit the enzyme by closely interacting with Glu151 residue. In general, electron-donating groups decrease G6PD enzyme activity and, conversely, electron-withdrawing groups decrease 6PGD enzyme activity. These rhodanines might become some future drug candidates for potent inhibitors of PPP enzymes.

**Figure 30:**
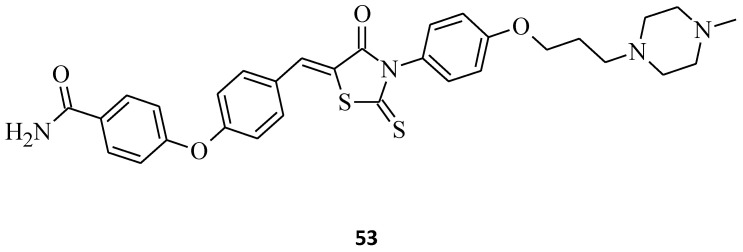
The structure of potent and selective rhodamine-type IKKβ inhibitor.

NF-ĸB is a multipurpose transcription factor that plays the main regulatory role of the genes related to inflammation, proliferation, and anti-apoptosis. The phosphorylation process of IĸB is catalyzed by the IĸB kinase complex protein (IKK), which consists of two central catalytic subunits, IKKα (IKK1), IKKβ (IKK2), and one regulatory IKKγ (NEMO). Both IKKα and IKKβ are serine-threonine kinases, but IKKα is believed to regulate the time of the NF-ĸB response with an extended expression of the proinflammatory cytokines spotted in IKKα-deficient cells. That is why the development of IKKβ selective inhibitors over IKKα is beneficial to autoimmune diseases such as cancer [60]. Structure **53** (Figure 30) was found to possess the highest inhibitory activity, with an IC_50_ value of 0.35 µM as well as excellent selectivity against IKKβ over other kinases such as IKKα, JNK1, JNK2, or JNK3. Both the NF-ĸB activation and the TNFα production were successfully blocked by compound **53** [60]. The results of the cell-based assay indicated that the IKKβ inhibitory activities were influenced mostly by amino groups in the western part of the rhodanine ring and the location of carboxamido substituent in the eastern part of the derivatives. These findings may suggest that rhodanine derivatives with aminoalkoxy substituents, such as the molecule **53**-bearing 4-methylpiperazinylpropoxyphenyl group in the western part and the *para*-carboxamidophenoxyphenyl moiety in the eastern part of the rhodanine nucleus, could become potential candidates for the treatment of the diseases linked with NF-ĸB activation, such as cancer, as effective IKKβ inhibitors.

**Figure 31:**
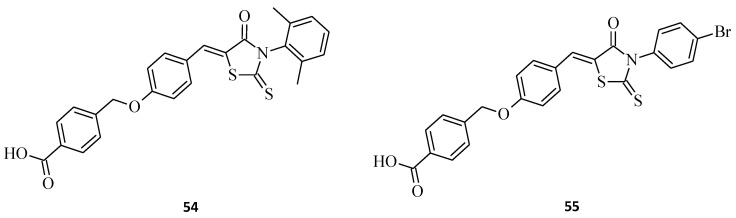
The structures of 3-aryl-rhodanine benzoic acids as anti-apoptotic protein Bcl-2 inhibitors.

Resistance to the conventional therapies of human cancer often results from the evasion of apoptosis, which is characteristic of malignancies. Therefore, targeting essential apoptosis regulators is a promising strategy for developing potent therapeutic agents to improve their treatment. The Bcl-2 (B-cell lymphocyte/leukemia-2) family proteins are the main factors that regulate the process of apoptosis and the composition of anti-apoptotic proteins, such as Bcl-2, Bcl-xL, Mcl-1, Bfl-1/A1, Bcl-B, and Bcl-w, and pro-apoptotic proteins, including BAK, BAX, BID, BIM, and BAD [61]. Studies are proving that the anti-apoptotic Bcl-2 proteins tend to be overexpressed in various types of human cancers, including B-cell lymphomas [62], breast carcinomas [63], and prostate cancers [64]. The available data also indicate their contribution to cancer initiation and progression, as well as their resistance to some of the current anticancer treatments [65]. Small-molecule inhibitors, including 2-thioxo-4-thiazolidinone-based derivatives, have been reported as effective Bcl-2 family inhibitors. For example, BH3I-1 induced apoptosis by binding to the BH3 site of the anti-apoptotic Bcl-2 proteins [66], and WL-276, as its preliminary biological activity assay, indicated the possibility of tumor growth suppression [67]. Huansheng Fu et al. [68] developed a new compound possessing a 3-aryl-rhodanine benzoic acid structure that inhibited Bcl-2 protein by 18% at 100 µM and then designed novel rhodanine derivatives based on this molecule. Synthesized compounds **54** and **55** displayed the best Bcl-2/Mcl-1 inhibitory activities with the binding affinities below 1 µM (Figure 31). The strong affinities of structures **54** and **55** indicate that *para*-bromophenyl and *ortho*-, *para*-dimethoxyphenyl substituents on the 3-position of the rhodanine ring benefit from the higher potency of the compounds. Interestingly, the molecules had much better activities when compared to the initial structure. Furthermore, both of the compounds displayed Bcl-2/Mcl-1 selectivity over Bcl-xL. These results suggest that rhodanine-based benzoic acid derivatives could become lead structures for designing potent and Bcl-2/Mcl-1 selective inhibitors.

**Figure 32:**
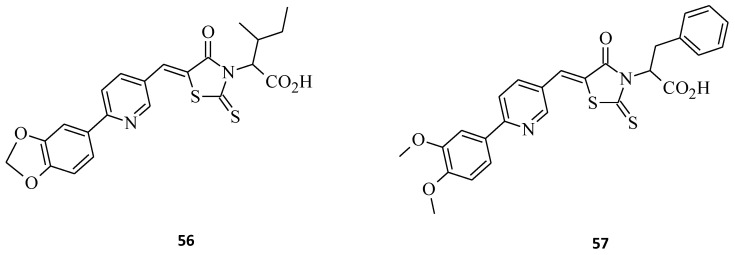
The structures of rhodanine-based compounds with binding activity against Bcl-XL and Mcl-1.

Referring to the compound BH3I-1, as a well-known inhibitor of the Bcl-2 proteins [69], its modifications can result in different binding profiles to Bcl-xL protein, with an increase in molecule efficacy [70,71]. Bernardo et al. [72] developed novel pyridylrhodanines and, amongst them, structures **56** and **57** as potential inhibitors of Bcl-xL and Mcl-1 (Figure 32). Compound **56** showed the best binding and selectivity towards Bcl-xL (*K*_i_= 3.6 µM), whereas **57** was the most selective binder with the respect to the Mcl-1 protein (*K*_i_= 8.5 µM). Interestingly, structure **57,** despite the strong affinity to Mcl-1 had no observed binding towards Bcl-xL.

**Figure 33:**
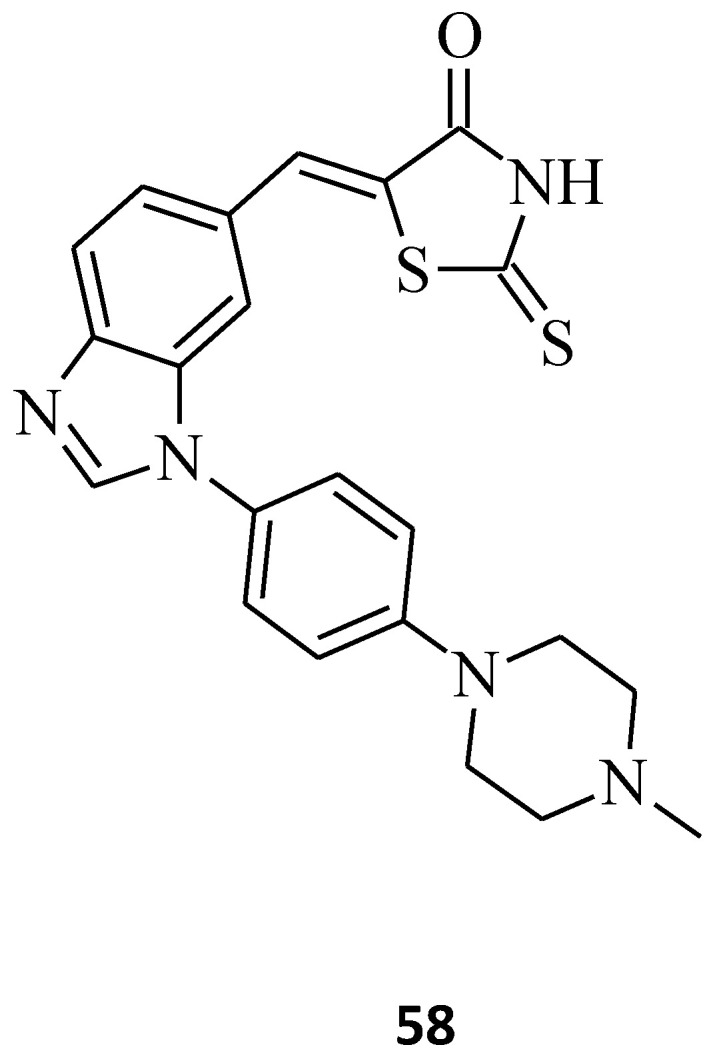
The structure of a potent Pan-Pim kinases inhibitor with rhodanine-benzimidazole moiety.

The Pim kinase family members consist of Pim-1, -2, and -3, which are highly homologous to each other [73]. The serine/threonine Pim kinases are overexpressed in different types of solid carcinomas and hematological malignancies and contribute to regulating cell-cycle progression and cell survival [74]. Moreover, Pim kinases were suggested to take part in angiogenesis and anticancer drug resistance in chemotherapy [75]. Sawaguchi et al. found a potent and selective Pim kinases inhibitor, compound **58**, with a rhodaninebenzoimidazole structure (Figure 33) [76]. Compound **58** inhibited Pim-1, -2, and -3, with IC_50_ values of 16, 13, and 6.4 nM, respectively. This molecule, with a 1*H*-benzo[*d*]imidazole ring and methylpierazine as an aliphatic amine through the phenyl group as a linker, suppressed the proliferation of solid and hematological cancer cell lines at submicromolar concentrations. The given data suggest that compound **58** can serve as a lead to new anticancer agents which are effective in the treatment of both solid carcinomas and hematological malignancies.

**Figure 34:**
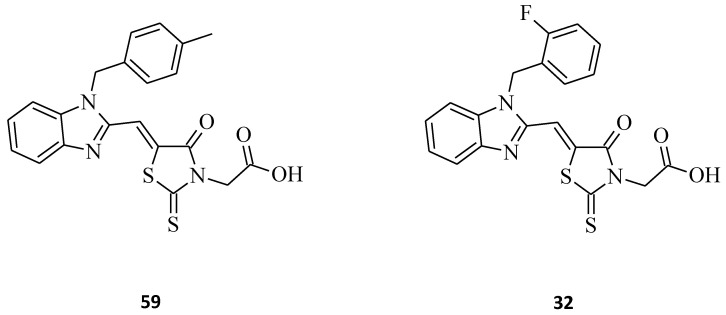
The structures of potent benzimidazole-rhodanine conjugates as topoisomerase II inhibitors.

DNA topoisomerases are the main cellular enzymes found in nearly all kinds of living cells. These enzymes mediate DNA replication, repair, transcription, recombination, and chromatin assembly [77,78]. Certain of the most effective anticancer drugs, such as etoposide, doxorubicin, or amsacrine, have been reported as Topo II inhibitors [79]. Although these compounds tend to exhibit some serious side effects during chemotherapy, which limit their therapeutic values, the development of novel, potent drugs such as Topo II inhibitors is necessary for improving the quality of cancer treatment [80,81,82]. Some recent structure–activity relationship studies showed that the benzimidazole ring introduced to the structures as the fused system is important for Topo II inhibitory potency, as is the phenyl group linked to this moiety [83]. Mechanism studies supported by molecular docking revealed that these molecules block the ATP-binding site of the enzyme [84,85]. Penghui Li et al. synthesized benzimidazole-rhodanine conjugates **59** and **32** (Figure 34) and evaluated them for their Topo I and II inhibitory properties [32]. The tested compounds turned out to be non-intercalating Topo II catalytic inhibitors, showing strong inhibitory activities at 10 μM. Both rhodanine derivatives, **59** with 2-fluorobenzyl and **32** with 4-methylbenzyl substituted benzimidazole moieties, indicate that the rhodanine ring and the phenyl group are particularly significant for the Topo II inhibitory potency.

**Figure 35:**
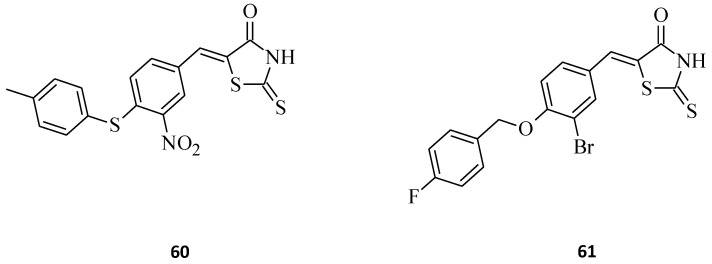
The structures of rhodanine derivatives, potent as inhibitors of human DNA polymerase λ.

Human DNA polymerase λ (DNA Pol λ) is a key enzyme for maintaining the genetic integrity of the genome. The rhodanines, which are an excellent drug scaffold, were found to be the most potent inhibitors for DNA Pol λ. DNA Pol λ can synthesize DNA in a template-dependent manner, *de novo*, and possesses terminal deoxynucleotidyl transferase (TDT) activity [86,87]. An investigation of the expression patterns of specialized DNA polymerases in 68 different tumor samples revealed that in more than 45% of these tumors at least one specialized DNA polymerase was 2-fold-enhanced expressed [88]. Strittmatter et al. [35], in their work, focused on the recently described human DNA polymerase λ (DNA Pol λ), a member of the DNA polymerase X family [89]. One goal for targeting these DNA polymerases is the inhibition of the repair of DNA adducts caused by DNA-damaging anticancer agents. Known inhibitors of the polymerase function of DNA Pol λ are exclusively based on natural products [90]. Three classes of compounds were analyzed towards inhibiting the DNA polymerase function of DNA Pol β [35]. Class I was rhodanines, namely 5-arylidene-2,4-thiazolidinediones, class II consisted of carbohydrazides, and class III contained a common 2,4-pentadione substructure element. The rhodanines, being an excellent scaffold for the developing biologically active molecules [91], inhibited the polymerase function of DNA Pol λ. These compounds were able to discriminate between DNA Pol λ and β, where compound **60** (Figure 35) was the most potent discriminating inhibitor. It was found that structure **60** dose-dependently inhibits the polymerization function of DNA Pol λ, with an IC_50_ value of 5.9 μM, and DNA Pol β, with an IC_50_ of 64.4 μM, and hence could discriminate between the two highly similar families of X DNA polymerases with a factor of ∼10. These data indicate that the rhodanine moiety is very important for a highly active inhibitor. Rhodanines are nonmutagenic [92], and a long-term study was conducted on their clinical effects on compounds such as, for example, rhodanine-based epalrestat, which was well tolerated by patients [93], while DNA Pol λ was discussed as a promising cellular target, especially in the case of cancer treatment [94]. The half-maximal inhibitory concentration of the cell viability was determined (EC_50_) for the discovered rhodanines, including compounds **60** and **61** (Figure 35), using two human cancer cell lines, a cervix carcinoma cell line, HeLa S3, and a hepatocellular carcinoma cell line, Hep G2 [35]. In both of these cancer types, DNA Pol λ is overexpressed [95].

**Figure 36:**
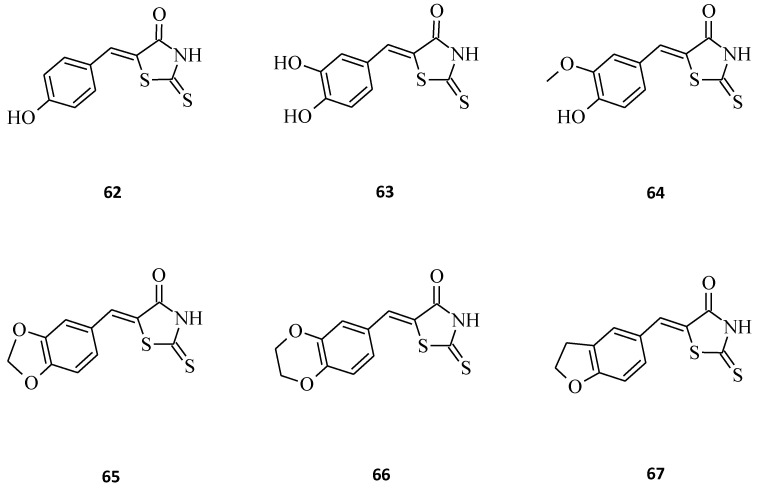
The structures of the novel (5*Z*)-5-arylidene-2-thioxo-1,3-thiazolidin-4-one derivatives as inhibitors of protein kinase DYRK1A.

Among the 538 human kinases, DYRKs (dual-specificity tyrosine phosphorylation regulated kinases, consisting of 5 members) is a family of eukaryotic kinases that are associated with a larger CMGC family of proline/arginine-directed serine/threonine kinases. In this DYRK family, there are five mammalian subtypes (1A, 1B, 2, 3, and 4). The Dyrk1A gene is located within the human chromosome 21 Down Syndrome Critical Region (DSCR) [96]. According to recent literature, DYRK1A occurs due to its involvement in different diseases, including Alzheimer’s disease (AD), Down syndrome (DS) [97], and cancer [98,99,100]. Bourahla et al. [101] designed a series of novel compounds, including (5*Z*) 5-arylidene-2-thioxo-1,3-thiazolidin-4-one derivatives prepared under microwave irradiation from various aromatic aldehydes and respective 2-thioxo-1,3-thiazolidin-4-ones, and some valuable results for structures **62**, **63**, **64**, **65**, **66**, and **67** were obtained (Figure 36). Compound **62**, with a hydroxyl group at the C-4 position of the exocyclic phenyl moiety, exhibited a sub-micromolar inhibitory effect towards DYRK1A (IC_50_ = 0.028 μM). Interestingly, compound **63**, bearing a supplementary hydroxyl group at the C-3 position of the phenyl ring, was completely inactive (IC_50_ > 10 μM), which indicates that the presence of only a single hydroxyl group on the phenyl moiety seems to be essential for an optimal inhibitory effect. In structure **64**, the introduction of a small methoxy group resulted in the DYRK1A inhibition activity decrease (IC_50_ = 0.064 μM). The introduction of more bulky groups at the 5-ylidene position in **65**, **66**, and **67**, as 1,3-benzodioxol-5-yl, 2,3-dihydro-1,4-benzodioxin-5-yl, or 2,3-dihydro-benzofuran-5-yl, respectively, resulted in the maintenance of sub-micromolar kinase inhibitory activity.

**Figure 37:**
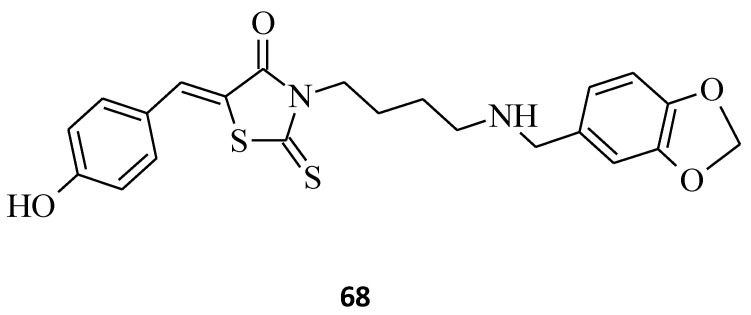
The structure of 3-(4-arylmethylamino)butyl-5-arylidene-rhodanine displaying inhibition activity on *Ss*CK1.

Casein kinase 1 (CK1) is a monomeric serine-threonine protein kinase with seven isoforms: α, β, γ1, γ2, γ3, δ and ε. CK1 is involved in many cellular processes, including DNA repair, cell division, nuclear localization, and membrane transport. Isoforms are also integral to development [102]. For example, compound **68** (Figure 37) exhibited a promising inhibitory effect on *Ss*CK1 (IC_50_ values for **68**: 1.4 μM) with good selectivity. These results may be the starting point for a new, larger group of 3-(4-Arylmethylamino)butyl-5-arylidene-rhodanine derivatives and further investigation of the biological properties of these novel porcine casein kinase 1, *Ss*CK1 inhibitors with potential applications in cancer [40].

## 4. Conclusions

In summary, this article provides an overview of the information about the anticancer activity of rhodanines published in the last decade. The rhodanine heterocycle is a privileged core in medicinal chemistry and is highly effective in many kinds of biological activity. This review describes the structure–activity relationship and some molecular targets for rhodanine derivatives. 

The rhodanine derivatives showed great potential as anticancer agents, and some of them demonstrated activity in the range of micromolar concentration (0.2–0.6 µM) as well as revealing a good safety profile. The results of the structure–activity relationship analysis demonstrated that the presence of hydrogen donor groups, such as carboxyl or phenol hydroxyl connected with a small linker in position 3 of rhodanine, was more beneficial for anticancer activity than their more bulky homologues. In addition, the presence of heteryl moiety in position 5 of the 2-thioxothiazolidin-4-one ring was also better for anticancer activity in comparison with the aryl substituents. The structure–activity relationship analysis also suggested that 3,5-disubstituted rhodanine derivatives generally showed better anticancer potential than their 3- or 5-monosubstituted precursors. 

Therefore, this review appears to be important for the further development of the rational drug design of new candidates with anticancer potential among rhodanine derivatives and their structural analogues.

## Supplementary Materials

The following supporting information can be downloaded at: https://www.mdpi.com/article/10.3390/molecules27123750/s1, Table S1: The most potent 3- or 5-substituted, and 3,5-disubstituted rhodanine derivatives as anticancer agents.

## Abbreviations

6PGD—6-phosphogluconate dehydrogenase; A2780—human ovarian carcinoma cell line; A2780cisR—cisplatin-resistant human ovarian carcinoma cell line; A549—adenocarcinomic human alveolar basal epithelial cells; AG01523—normal human skin fibroblasts; AGS—gastric cancer cell line; BAK, BAX, BID, BIM, and BAD—pro-apoptotic proteins; Bcl-2—B-cell lymphoma 2, protein that regulates cell death (apoptosis); Bcl-B—anti-apoptotic protein; Bcl-w—anti-apoptotic protein; Bcl-xL—anti-apoptotic protein; Bfl-1/A1—anti-apoptotic protein; BH3I-1—selective inhibitor of Bcl-2 family proteins; Caco-2—human colon adenocarcinoma cell line; CCRF-CEM—lymphoblastic leukemia cell line; CK1—casein kinase 1; CMGC—kinases family, cyclin-dependent kinase (CDK), mitogen-activated protein kinase (MAPK), glycogen synthase kinase (GSK) and CDC-like kinase (CLK); CTC_50_—50% of cytotoxicity inhibition; Dami—megakaryoblastic leukemia cell line; DLD-1—human colon cancer cell line; DNA Pol β—DNA polymerase beta; DNA Pol λ—human DNA polymerase λ; DSCR—Down syndrome critical region; DU-145—human prostate cancer cell line; DYRK1A—dual specificity tyrosine phosphorylation regulated kinase 1A; DYRKs—dual-specificity tyrosine phosphorylation regulated kinases; EC_50_—half-maximal inhibitory concentration of the cell viability; EGFR—epidermal growth factor receptor; G6PD—glucose-6-phosphate dehydrogenase; GI_50_—molar concentration of the compound that inhibits 50% net cell growth; GPmean—mean growth percentage; H460—lung cancer cell line; HaCat—human epidermal keratinocyte cell line; HCT 116—human colorectal carcinoma cell line; HeLa—cervical cancer cells; HeLa S3—cervix carcinoma cell line; Hep—Hep G2 hepatocellular carcinoma cell line; HGC—human gastric cancer cell line; HL-60—human leukemia cell line; HOP-92—non-small cell lung cancer cell line; HT-1080—human fibrosarcoma cell line; HT29—human colorectal adenocarcinoma cell line; Huh7 D12—hepatocellular carcinoma cell line; IC_50_—half-maximal inhibitory concentration; IKK—kinase complex protein; IKKα—(IKK1) central catalytic subunit; IKKβ—(IKK2) central catalytic subunit; IKKγ—(NEMO) central regulatory subunit; inhibitory %—inhibition of particular cancer cell line growth in percent; IκB—kinase (IKK), an enzyme complex that is involved in propagating the cellular response to inflammation; JNK1, JNK2 or JNK3—c-Jun N-terminal kinases; K562—chronic myelogenous leukemia cell line; *K*_i_—dissociation constant describing the binding affinity between the inhibitor and the enzyme; MCF-7—human breast cancer cell line; Mcl-1—anti-apoptotic protein; MDA-MB-231—human breast cancer cell line; MNK 74—gastric cancer cell line; MOLT-4—lymphoblastic leukemia cell line; NF-ĸB—multipurpose transcription factor; NF-ĸB—protein complex that controls transcription of DNA, cytokine production and cell survival; NRK-52E—normal rat kidney cell line; PI3K—phosphatidylinositol 3-kinase; Pim-1, 2, 3—Pim kinase family; PPP—pentose phosphate pathway; PRL-3—phosphatase of regenerating liver 3; PRLs—phosphatase of regenerating liver family; PTP4A—protein tyrosine phosphatase 4A; Raji—human lymphoma cancer cell line; RPMI-8226—human plasmacytoma cell line; SF-539—central nervous system tumor cell line; SK-MEL-5—melanoma cell line; SR—human lymphoma cell line; *Ss*CK1—porcine casein kinase 1; SW-620—colon cancer cell line; TDT—terminal deoxynucleotidyl transferase; TNFα—tumor necrosis factor; Topo II—topoisomerase II; WL-276—small-molecule antagonist against antiapoptotic Bcl-2 family proteins.
