# Supplementary material for: Anticancer Profile of Rhodanines: Structure–Activity Relationship (SAR) and Molecular Targets—A Review
Source: Molecules. 2022 Jun 10;27(12):3750. doi: 10.3390/molecules27123750 (PMC9231410; doi:10.3390/molecules27123750)
Supplement: Supplementary file 1 [file molecules-27-03750-s001.zip › molecules-1677895-supplementary.pdf]

Supplemental materials for

Anticancer profile of rhodanines: structure-activity relationship (SAR) and molecular targets. A review

Jacek Szczepański, Helena Tuszewska and Nazar Trotsko *

Chair and Department of Organic Chemistry, Faculty of Pharmacy, Medical University of Lublin, 4a Chodźki Street, 20-093 Lublin, Poland; jaacek.szczepanski.93@gmail.com (J.S.); tuszewska.helena93@gmail.com (H.T.); nazar.trotsko@umlub.pl (N.T.)

* Correspondence: nazar.trotsko@umlub.pl (N.T.); Tel.: +48 81 448 7244

Table S1. The most potent 3-or 5-substituted, and 3,5-disubstituted rhodanine derivatives as anticancer agents

Nr.	Structure	Cell lines	IC_{50}, % inhibition	References
**1**	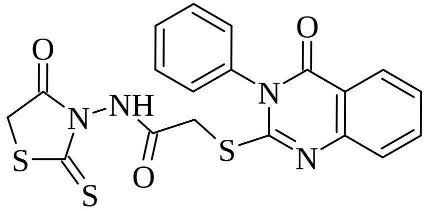	MCF-7	64.4%	[25]
**2**	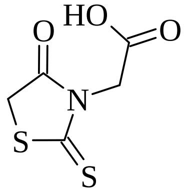	K-562	14.6 μg/mL	[26]
**3**	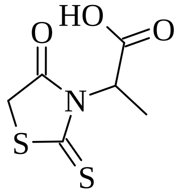	K-562, HeLa	11.1 μg/mL, < 200 μg/mL	[25,26]
**4**	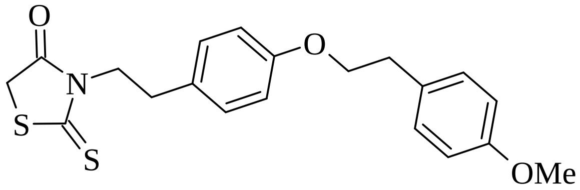	HCT 116	10 μM	[28]

**5**	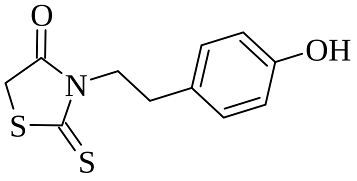	MDA-MB231,<br>HCT 116,<br>Caco 2	2 μM,<br>2 μM,<br>3 μM	[28]
**6**	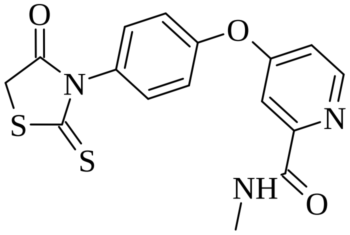	A549	43.6 μM	[12]
**7**	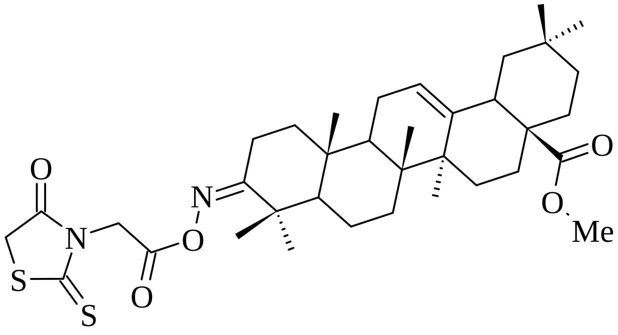	CCRF-CEM/L	56.34%	[29]
**8**	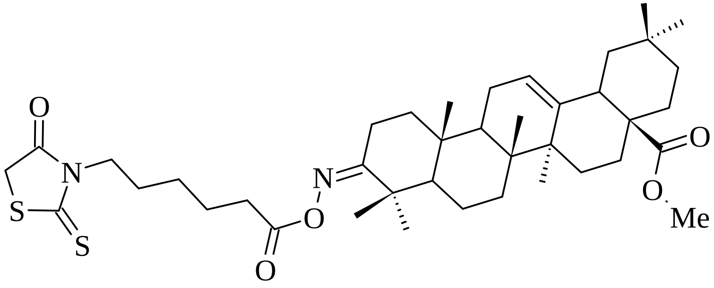	T-47D/BC	76.91%	[29]

**9**	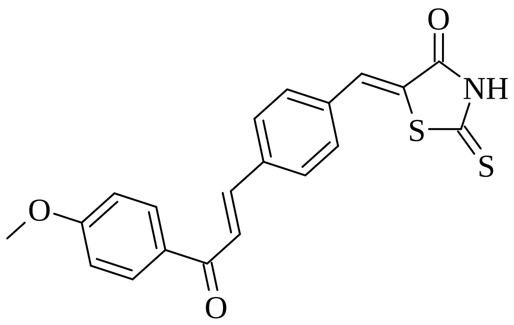	HeLa,<br>HT29,<br>A549,<br>MCF-7	28.3 μM,<br>24.5 μM,<br>26.6 μM,<br>28.6 μM	[11]
**10**	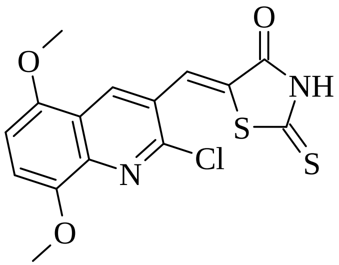	HGC,<br>DU-145,<br>MCF-7	7.2 μM,<br>15.3 μM,<br>9.5 μM	[30]
**11**	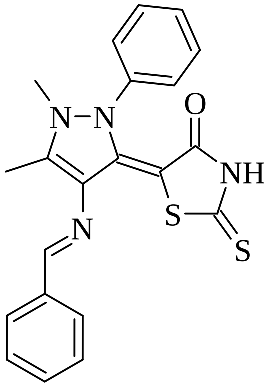	MCF-7	7.67 μg/mL	[34]

**12**	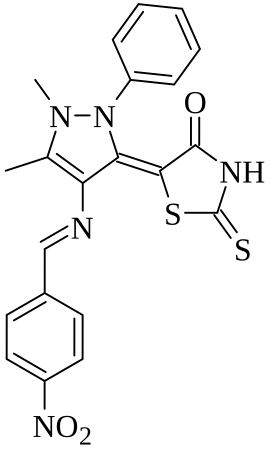	MCF-7	11.7 μg/mL	[34]
**13**	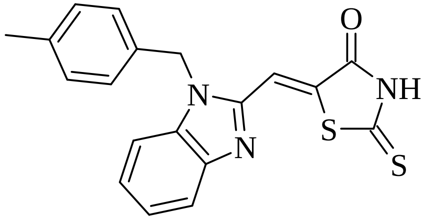	HL-60,<br>A549,<br>Raji,<br>MDA-MB201	2.66 μM,<br>5.31 μM,<br>4.48 μM,<br>6.42 μM	[35]
**14**	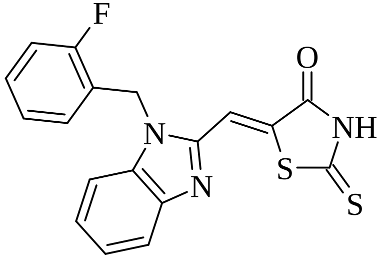	HL-60,<br>A549,<br>Raji,<br>MDA-MB201	8.42 μM,<br>> 50 μM,<br>25.48 μM,<br>21.33 μM	[35]

**15**	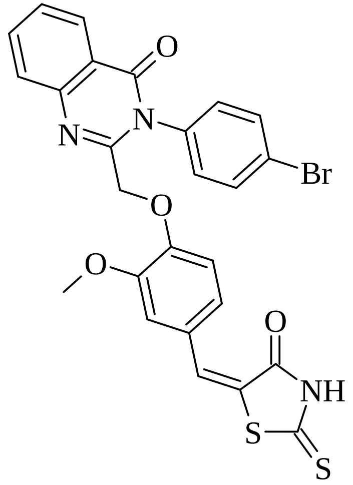	HT-1080,<br>HL-60,<br>K-562,<br>AG01523	8.7 μM,<br>1.2 μM,<br>1.5 μM,<br>> 100 μM	[36]
**16**	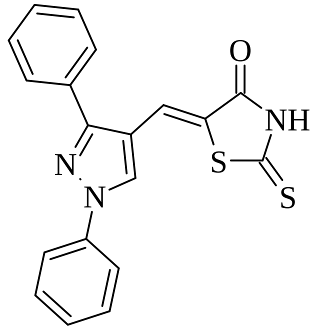	CCRF-CEM,<br>RPMI-8226,<br>HOP-92,<br>Normal human cell line	GI_{50}= 2.50 μM,<br>GI_{50}= 2.52 μM,<br>GI_{50}= 0.62 μM,<br>LC_{50}> 100 μM	[32]
**17**	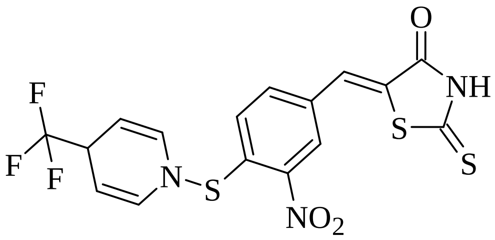	HeLa S3,<br>Hep G2	EC_{50}= 7.9 μM,<br>EC_{50}= 6.1 μM	[33]

**18**	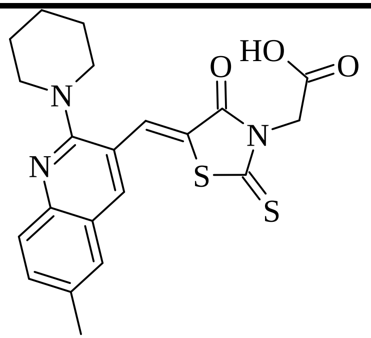	HGC, MNK-74	11.2 μM, 13.9 μM	[30]
**19**	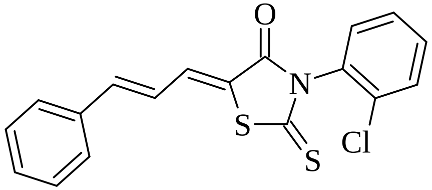	MCF-7	81%	[36]
**20**	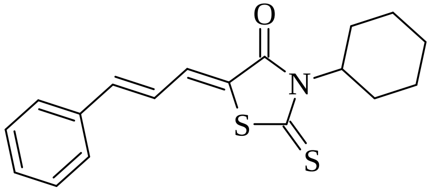	MCF-7	77%	[36]
**21**	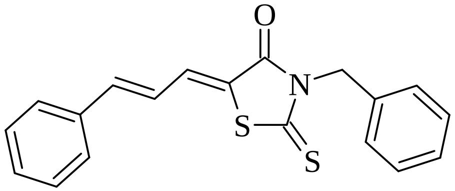	MCF-7	71%	[36]

**22**	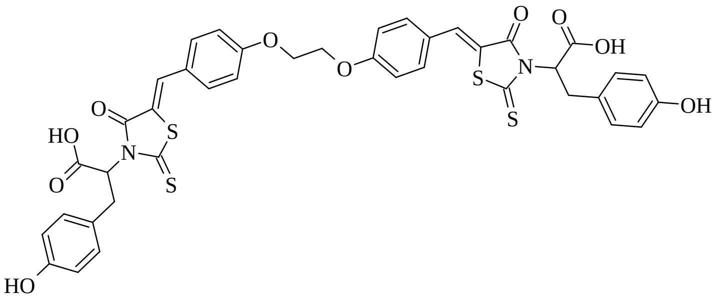	A549	CTC_{50}= 3.6 μg/mL	[37]
**23**	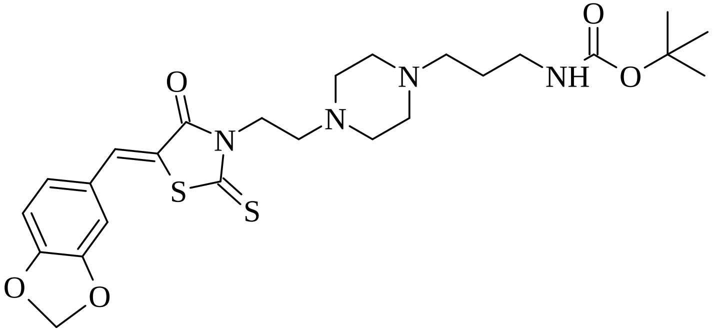	Huh7 D12,<br>Caco 2	8 μM,<br>8 μM	[13]
**24**	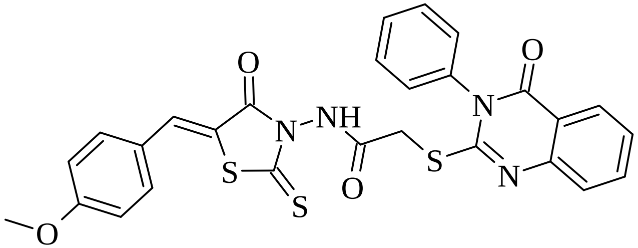	MCF-7	82.5%	[25]
**25**	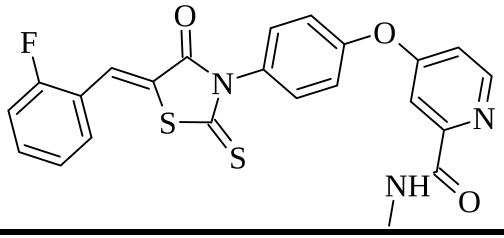	A549,<br>H460,<br>HT29	0.8 μM,<br>1.3 μM,<br>2.8 μM	[12]

**26**	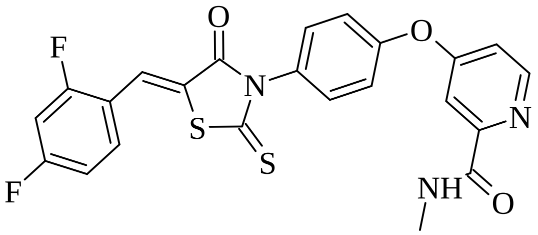	A549,<br>H460,<br>HT29	3.1 μM,<br>0.3 μM,<br>2.8 μM	[12]
**27**	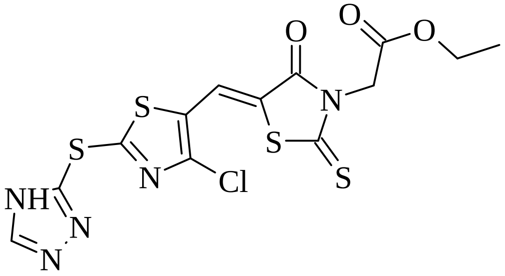	Huh7,<br>MCF-7	4.67 μM,<br>2.30 μM	[38]
**28**	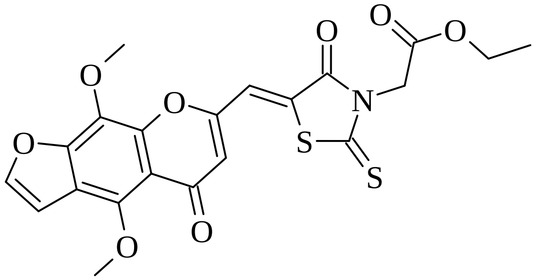	MCF-7,<br>MDA-MB-231,<br>Huh7	EC_{50}= 1.732 μM,<br>EC_{50}= 2.912 μM,<br>EC_{50}= 3.797 μM	[39]

**29**	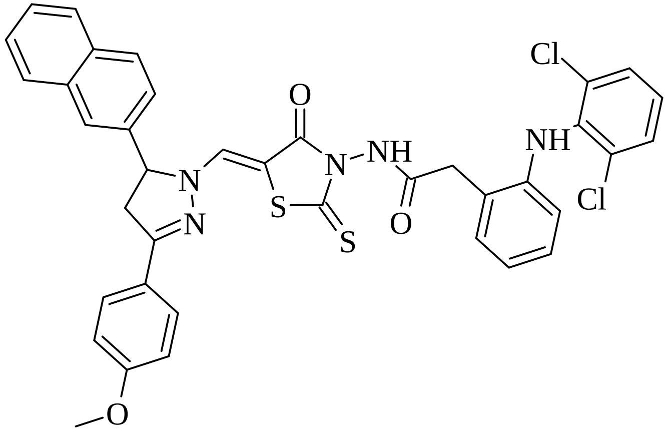	panel of approximately sixty cancer cell lines[1]	GP mean= 22.40%	[10]
**30**	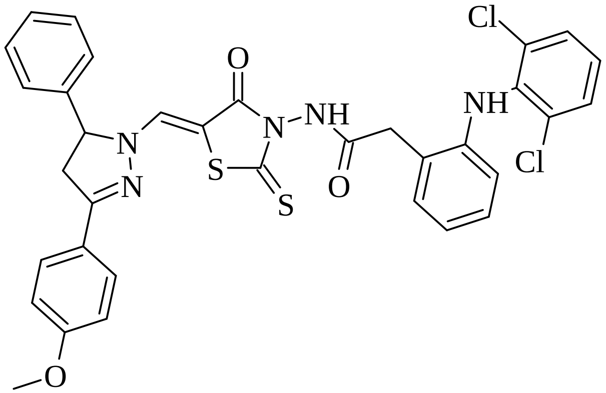	panel of approximately sixty cancer cell lines[1]	GP mean= 99.30%	[10]
**31**	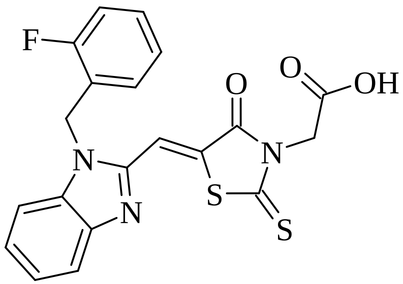	HL-60,<br>MDA-MB-201,<br>Raji,<br>A549	0.21 μM,<br>0.33 μM,<br>1.23 μM,<br>2.67 μM	[35]

**32**	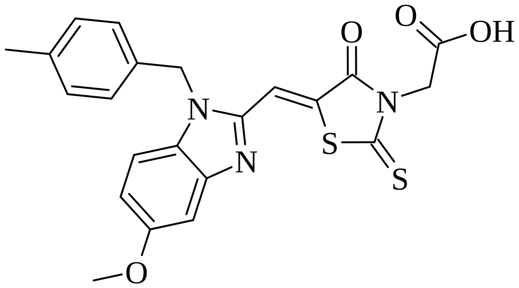	HL-60,<br>MDA-MB-201,<br>Raji,<br>A549	0.77 μM,<br>1.22 μM,<br>1.79 μM,<br>5.68 μM	[35]
**33**	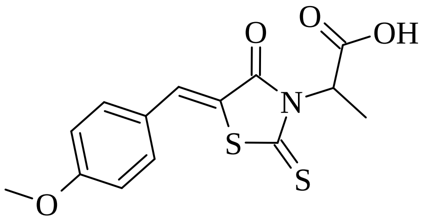	HeLa	52.00%	[27]
**34**	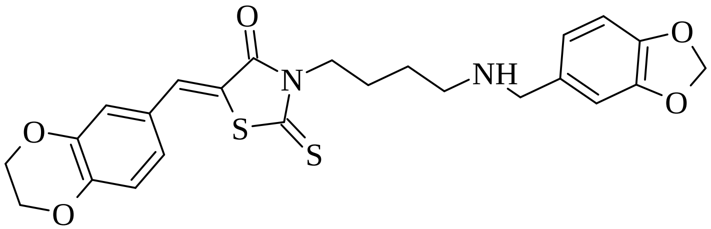	Huh7 D12,<br>HaCat,<br>MDA-MB231,<br>normal fibroblasts	9 μM,<br>8 μM,<br>9 μM,<br>> 25 μM	[40]
**35**	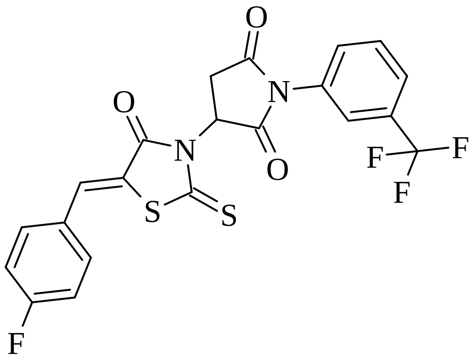	Dami	35.10%	[41]

**36**	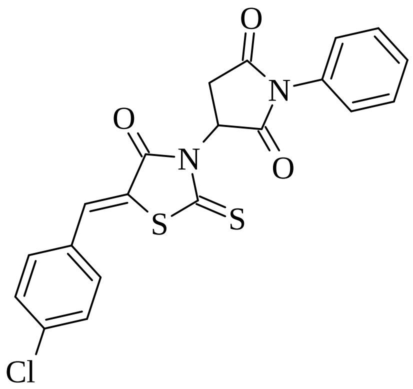	Dami,<br>HL-60	44.75%,<br>40.30%	[41]
**37**	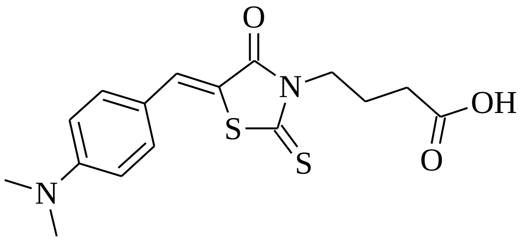	A2780,<br>A2780cisR	4.4 nM,<br>3.3 nM	[42]
**38**	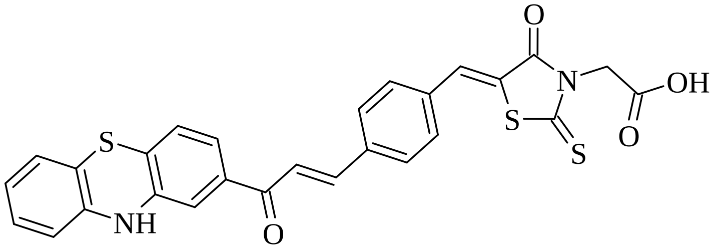	K562,<br>K562	8.79 μg/mL - T.B. assay,<br>19.62 μg/mL - MTT assay	[43]
**39**	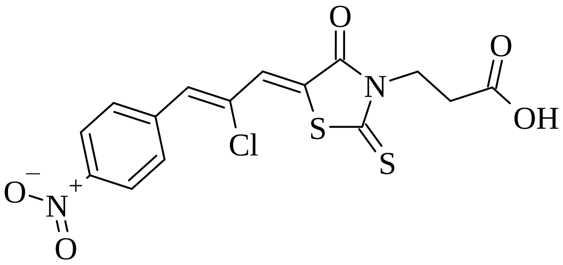	60 tumor cell lines MG_MID[2]	GI_{50}= 1.57 μM	[44]

**40**	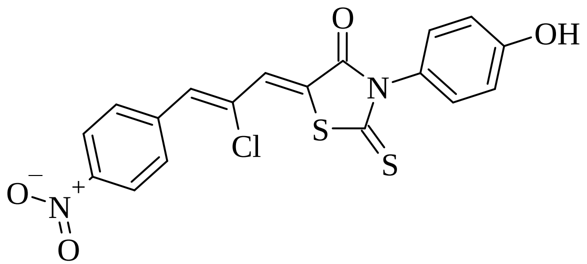	60 tumor cell lines MG_MID[2]	GI_{50}= 2.8 μM	[44]
**41**	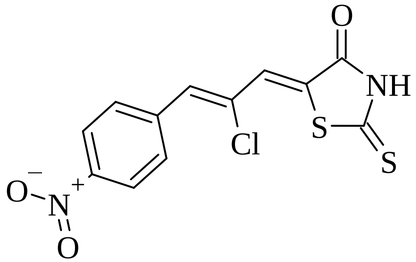	60 tumor cell lines MG_MID[2]	GI_{50}= 4.79 μM	[44]
**42**	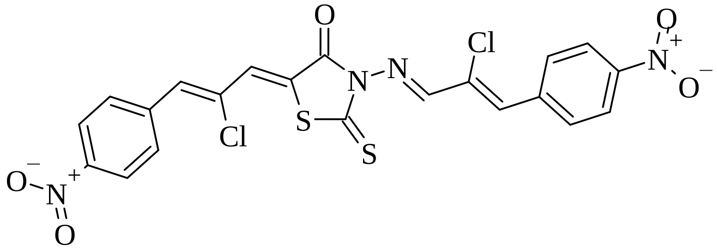	60 tumor cell lines MG_MID	GI_{50}= 18 μM	[44]
**43**	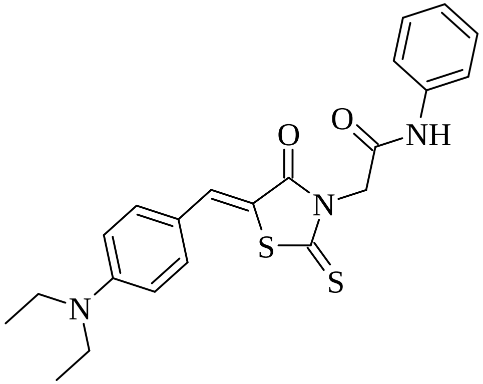	A549,<br>NRK-52E	7 μM,<br>14.7 μM	[46]

**44** 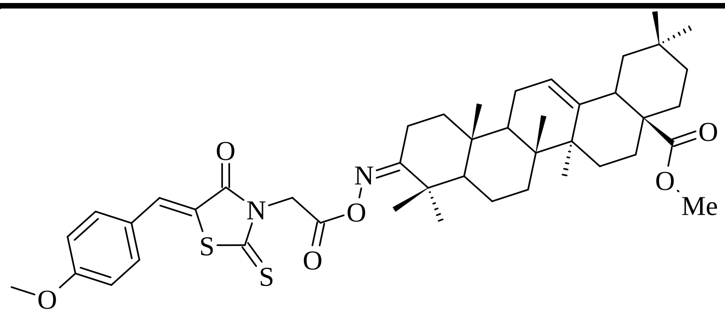	BT-549/BC	67.36%	[29]
**45** 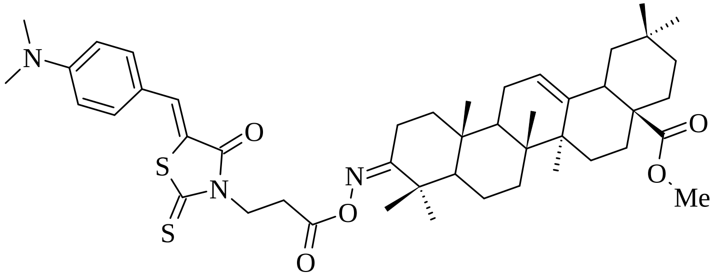	IGROV1/OC	82.21%	[29]

MG_MID - mean graph midpoint, arithmetical mean value for all tested cell lines.

[1,2] panel of approximately sixty cancer cell lines and 60 tumor cell lines MG_MID the next cell lines were used: leukemia (CCRF-CEM, HL-60, K562, MOLT-4, RPMI-8226, SR), NSC lung cancer (A549, EKVX, HOP-62, HOP-92, NCI-H266, NCI-H23, NCI-H322M, NCI-H460, NCI-H522), colon cancer (COLO 205, HCT 116, HCT-15, HT29, KM12, SW-620, HCC-2998), CNS cancer (SF-268, SF-295, SF-539, SNB-19, SNB-75, U251), melanoma (LOX IMVI, MALME-3M, M14, MDA-MB-435, UACC-62, UACC-257, SK-MEL-2, SK-MEL-5, SK-MEL-28), ovarian cancer (IGROVI, OVCAR-3, OVCAR-4, OVCAR-5, OVCAR-8, NCI/ADR-RES, SK-OV-3), renal cancer (786-0, A498, ACHN, CAKI-1, RXF 393, SN12C, TK-10, UO-31), prostate cancer (PC-3, DU-145), breast cancer (MCF-7, MDA-MB-231, HS 578T, BT-549, T-47D, MDA-MB-468).
